# Strain-Specific Properties and T Cells Regulate the Susceptibility to Papilloma Induction by *Mus musculus* Papillomavirus 1

**DOI:** 10.1371/journal.ppat.1004314

**Published:** 2014-08-14

**Authors:** Alessandra Handisurya, Patricia M. Day, Cynthia D. Thompson, Michael Bonelli, Douglas R. Lowy, John T. Schiller

**Affiliations:** 1 Laboratory of Cellular Oncology, National Cancer Institute, National Institutes of Health, Bethesda, Maryland, United States of America; 2 Lymphocyte Cell Biology Section, Molecular Immunology and Inflammation Branch, National Institute of Arthritis, Musculoskeletal and Skin Diseases, National Institutes of Health, Bethesda, Maryland, United States of America; 3 Division of Rheumatology, Internal Medicine III, General Hospital of Vienna, Medical University of Vienna, Vienna, Austria; Fred Hutchinson Cancer Research Center, United States of America

## Abstract

The immunocytes that regulate papillomavirus infection and lesion development in humans and animals remain largely undefined. We found that immunocompetent mice with varying H-2 haplotypes displayed asymptomatic skin infection that produced L1 when challenged with 6×10^10^ MusPV1 virions, the recently identified domestic mouse papillomavirus (also designated “MmuPV1”), but were uniformly resistant to MusPV1-induced papillomatosis. Broad immunosuppression with cyclosporin A resulted in variable induction of papillomas after experimental infection with a similar dose, from robust in Cr:ORL SENCAR to none in C57BL/6 mice, with lesional outgrowth correlating with early viral gene expression and partly with reported strain-specific susceptibility to chemical carcinogens, but not with H-2 haplotype. Challenge with 1×10^12^ virions in the absence of immunosuppression induced small transient papillomas in Cr:ORL SENCAR but not in C57BL/6 mice. Antibody-induced depletion of CD3^+^ T cells permitted efficient virus replication and papilloma formation in both strains, providing experimental proof for the crucial role of T cells in controlling papillomavirus infection and associated disease. In Cr:ORL SENCAR mice, immunodepletion of either CD4^+^ or CD8^+^ T cells was sufficient for efficient infection and papillomatosis, although deletion of one subset did not inhibit the recruitment of the other subset to the infected epithelium. Thus, the functional cooperation of CD4^+^ and CD8^+^ T cells is required to protect this strain. In contrast, C57BL/6 mice required depletion of both CD4^+^ and CD8^+^ T cells for infection and papillomatosis, and separate CD4 knock-out and CD8 knock-out C57BL/6 were also resistant. Thus, in C57BL/6 mice, either CD4^+^ or CD8^+^ T cell-independent mechanisms exist that can protect this particular strain from MusPV1-associated disease. These findings may help to explain the diversity of pathological outcomes in immunocompetent humans after infection with a specific human papillomavirus genotype.

## Author Summary

Infection with papillomaviruses can cause benign warts (papillomas) on skin and mucosae of humans and animals but also malignancies, especially anogenital carcinomas and, in genetically predisposed or immunocompromised individuals, cutaneous squamous cell cancers. Control and clearance of these viruses are thought to be mediated by the cellular immune system, however experimental determination for the necessary cellular effector(s) is lacking. The recently identified mouse papillomavirus (MusPV1, also designated “MmuPV1”) is known to induce papilloma formation on skin of immunodeficient mice. However, its pathogenesis in immunocompetent mice is unclear. Our study shows that in an immunocompetent setting, MusPV1 generally causes asymptomatic skin infections, but no lesion outgrowth. Visible papillomas were consistently observed after profound immunosuppression in some, but not other, strains of mice. By selective removal of distinct cellular immune populations and employing genetically modified mice, we could show that T cells are pivotal for controlling MusPV1 infection and disease. We further show that surprising differences in the T cell subsets are required for protection in different strains of immunocompetent mice. This implies that unanticipated effector mechanisms can control virus infection and pathogenesis in specific genetic backgrounds. The findings may help to explain the wide of range of pathologic outcomes of infection by a specific human papillomavirus type in immunocompetent people.

## Introduction

Papillomaviruses (PV) are DNA tumor viruses that infect stratified squamous epithelia of the skin and mucous membranes of humans and many other vertebrate species [1]. PV infections are species-restricted and region-restricted, in that only part of the skin and mucous membranes of the host species of a given PV is permissive for productive infection [2]. More than 150 human PV (HPV) genotypes (types) have been identified. These viruses can induce long-term infection that, depending on the virus type and its human host, may not cause lesions, may induce benign lesions (warts or papillomas), or may lead to the development of anogenital carcinomas, most notably cervical and oropharyngeal cancers. Certain cutaneous HPV types have also been implicated in the pathogenesis of some epidermal squamous cell cancers in genetically predisposed or immunocompromised individuals [3].

Although neutralizing antibodies against the viral capsid proteins are sufficient to prevent PV infections, cell-mediated immunity is generally thought to control the infections once they become established. For instance, individuals with underlying T cell deficiencies, but not B cell deficiencies, often have difficulties in controlling and clearing HPV-induced neoplasia [4]. However, it has been difficult to provide experimental support for this concept or to determine which particular subset(s) of immunocytes are responsible for these activities. Although studies of animal PVs, such as bovine PV (BPV), cottontail rabbit PV (CRPV), and canine PV, have contributed to our understanding of PV biology [reviewed in 5], the limited immunological reagents available for these species have hampered critical investigations of immune regulation of PV infection with these systems.

The recent identification of MusPV1 (also designated “MmuPV1”), which is the first domestic mouse PV, provides an excellent opportunity to investigate the immune mechanisms that control PV infection in a mammalian species whose immunology has been well characterized. MusPV1 was found initially in an inbred NMRI-*Foxn1^nu^/Foxn1^nu^* nude laboratory mouse colony [6]. These immunodeficient mice spontaneously developed papillomas at cutaneous surfaces near the mucocutaneous junctions of nose and mouth, from which the virus was isolated. Subsequent studies reported that papillomas could be induced by experimental infection with MusPV1 in immunodeficient B6.Cg-*Foxn1^nu^/Foxn1^nu^*, *Foxn1^nu^/Foxn1^nu^* and SCID SHO mice [7], [8]. We also determined that athymic NCr nu/nu (nude) mice could be infected with *in vitro* synthesized full-length genomic DNA of MusPV1, giving rise to non-regressing cutaneous papillomas from which high titers of authentic, infectious MusPV1 virions were isolated and serially passaged [9].

In contrast to these reports of MusPV1 in immunodeficient mice, the study of MusPV1 in immunocompetent mice has been limited. The initial report of MusPV1 noted that when cell-free extracts from papillomas in the immunodeficient mice were used to inoculate the dorsal skin of S/RV/Cri-*ba/ba* mice, which have an unknown mutation that results in thin short hair, they induced small papules at most injection sites. These lesions, which were not characterized further, took at least three weeks to develop, and regressed spontaneously by 8 weeks post-inoculation [6]. Subsequent efforts to induce lesions in C57BL/6J mice were unsuccessful [7]. Thus establishment and further characterization of MusPV1 infection and disease in an immunocompetent setting were warranted.

In this study we aimed at characterizing MusPV1 infection in different immunocompetent murine strains and sought to determine the key immunologic players primarily responsible for control of cutaneous PV infection and papilloma induction. Our results revealed asymptomatic MusPV1 infection in these immunocompetents and demonstrated that profound immunosuppression can render these strains that had various H-2 haplotypes susceptible to MusPV1-induced papilloma formation of the skin. This is reminiscent of infection with HPV of genus beta that also predominantly induces asymptomatic skin infections in immunocompetent individuals but can induce visible lesions after immunosuppression. The observed differences in the efficiency of papilloma outgrowth between the individual murine strains corresponded partially to their previously reported susceptibility to chemical-induced skin papillomatosis [summarized in 10], suggesting that there may be similarities between their relative susceptibility to papilloma formation induced by MusPV1 and to chemical carcinogens. We further provide clear experimental proof that T cell functions are required for control of PV infection and disease and reveal striking differences in the protective capacities of T cell subsets among murine strains, including CD4-mediated effector mechanisms.

## Results

### Cyclosporin (CsA) administration promotes MusPV1 infection and papilloma formation in distinct murine strains

Several inbred immunocompetent strains of mice, namely FVB/NCr, BALB/cAnNCr, DBA/2NCr, A/JCr, C57BL/6NCr (C57BL/6), 129S6/SvEv, C3H/HeJCr, and as well the outbred Cr:ORL SENCAR (SENCAR) were evaluated for their susceptibility to papilloma induction by MusPV1 (Table 1). The inbred strains have a range of H-2 haplotypes and a range of reported susceptibility to chemical carcinogen-induced skin papillomas (Table 1) [10]–[12]. DBA/2NCr and BALB/cAnNCr are both H2^d^, but vary in their sensitivity to chemical carcinogenesis, as is also true for the two H2^b^ strains, C57BL/6 and 129S6/SvEv. The SENCAR strain was included because it was selectively bred for high susceptibility to skin tumor induction by chemical carcinogens. All strains were infected with 6×10^10^ MusPV1 virions per animal on pre-scarified tail skin, using a previously optimized procedure for PV infection of mouse skin [9], [13], and followed for 4 months. During this period, no papilloma outgrowth was observed in any strain, although the same preparation and dose of MusPV1 virions consistently induced large papillomas in immunodeficient athymic NCr nude mice within one month of inoculation (data not shown).

**Table 1:** Summary of papilloma development under cyclosporin A treatment in different murine strains.

strains	haplotype	no. of papillomas; mean lesion length	E1∧∧∧E4 gene expression (mean ± SD)	sensitivity to chemical-induced skin carcinogenesis
Cr:ORL SENCAR	outbred	6/8; 8.2 mm	24.2±11.7	+++
FVB/NCr	H2^q^	8/8; 7.6 mm	29.9±3.6	+++
BALB/cAnNCr	H2^d^	5/8; 4.6 mm	3.2±1.1	+
DBA/2NCr	H2^d^	0/4	n.d.	++
A/JCr	H2^a^	4/8; 2.9 mm	4.1±1.8	+
C57BL/6	H2^b^	0/8	1.2±0.4	+/−

**Figure 1:**
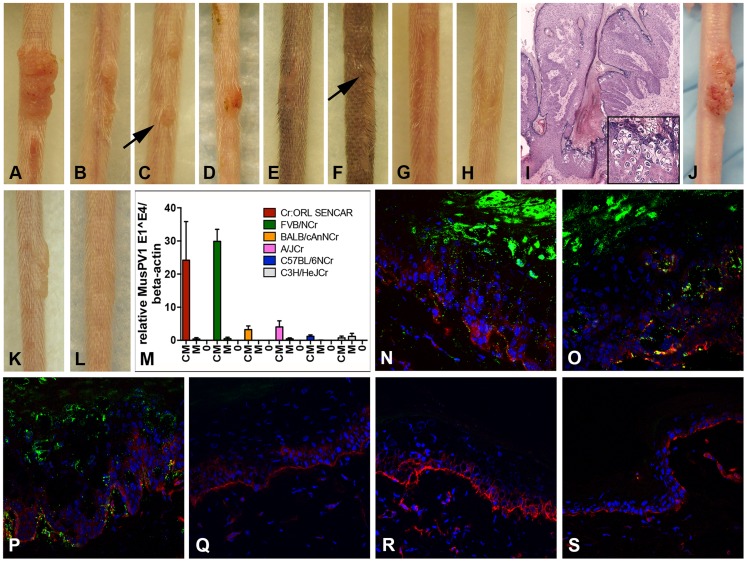
Cyclosporin (CsA) administration promotes MusPV1 infection and strain-dependent papilloma formation. CsA treatment was started one week prior to infection with 4.2×10^10^ MusPV1 virions per animal, and continued for a total of 5 weeks in several immunocompetent murine strains harboring different H-2 haplotypes. The treatment revealed a strain-dependent hierarchy of susceptibility: Highly susceptible (A) Cr:ORL SENCAR and (B) FVB/NCr; intermediate susceptible (C) BALB/cAnNCr and (D) A/JCr; resistant (E) C57BL/6NCr and (F) C3H/HeJCr mice. (G) MusPV1-infected Cr:ORL SENCAR control mice and (H) mock-infected/CsA-treated Cr:ORL SENCAR control mice did not develop any papillomas. (I) Hematoxylin-eosin stained tissue section taken from a papilloma derived from a CsA-treated/MusPV1-infected Cr:ORL SENCAR mouse (magnification 2.5×). The lesions showed histological features of papillomas with numerous koilocytes in the epithelium (inset). (J) MusPV1 virions that were extracted from lesions of CsA-treated/MusPV1-infected Cr:ORL SENCAR mice induced papilloma formation on the tail of an athymic NCr nude mouse after experimental transmission. (K) Florid papilloma on the tail of a CsA-treated/MusPV1-infected Cr:ORL mouse 4 weeks after infection. (L) Regressed lesion on the same mouse 6 weeks after cessation of CsA administration, showing that CsA treatment is required for induction and maintenance of the papillomas. (M) Determination of MusPV1-specific E1∧E4 spliced transcripts relative to endogenous beta-actin by real-time PCR 4 weeks post-infection showed that levels of MusPV1 E1∧E4 relative to beta-actin in skin tissues taken from CsA-treated/MusPV1-infected mice (**CM**), corresponded well to the lesion size. The levels of MusPV1 E1∧E4/beta-actin were low, but detectable in MusPV1-infected/untreated littermates (**M**). MusPV1 E1∧E4 spliced transcripts were undetectable in skin tissues of mock-infected mice (**0**). Evaluation of MusPV1 L1 protein expression in skin tissues taken 4 weeks post-infection by immunofluorescent staining revealed abundant MusPV1 L1 staining (green, detection with an Alexa Fluor 488-labeled secondary antibody) in the epithelium of (N) CsA-treated/MusPV1-infected Cr:ORL SENCAR, (O) CsA-treated/MusPV1-infected BALB/cAnNCr and (P) CsA-treated/MusPV1-infected C57BL/6NCr mice. L1 expression was punctate in the cytoplasm of the keratinocytes in the basal and lower spinous layers, and nuclear in the upper spinous and granular layers of the epithelium. L1 staining was undetectable in skin from (Q) MusPV1-infected Cr:ORL SENCAR, (R) MusPV1-infected BALB/cAnNCr and (S) MusPV1-infected C57BL/6NCr mice that had not received CsA. Basal keratinocytes were co-stained with a phycoerythrin-conjugated anti-CD49f antibody (red) to facilitate orientation. The epidermal compartment is shown in the upper part of each image and the dermal compartment in the lower part.

To determine whether immunosuppression renders these mouse strains susceptible to MusPV1-induced papilloma formation, animals were treated systemically with the immunosuppressant CsA, which can inhibit T cell activation and lymphokine production and is routinely used in humans for prevention of transplant rejection [14], [15]. CsA treatment was initiated one week prior to infection with 4.2×10^10^ MusPV1 virions and continued for additional 4 weeks, during which the mice were evaluated for papilloma formation. The results uncovered a hierarchy of susceptibility to MusPV1-induced papillomas among the strains (Table 1 and Figure 1A–H). SENCAR and FVB/NCr mice were highly susceptible. Most (6/8; 75%) of the SENCAR mice (Figure 1A) developed large and raised papillomas, with a mean length of 8.2 mm. All of the FVB/NCr mice (Figure 1B) inoculated with MusPV1 developed papillomas (8/8; 100%), which were moderately elevated, with a mean length of 7.6 mm. The BALB/cAnNCr, A/JCr, and 129S6/SvEv strains displayed intermediate susceptibility for papilloma development. For BALB/cAnNCr (Figure 1C), 5/8 animals developed papillomas, which were smaller than those in SENCAR and FVB/NCr, with a mean length of 4.6 mm. The comparable numbers for A/JCr mice (Figure 1D) were 4/8 and 2.9 mm, respectively, while those for 129S6/SvEv (Figure S1A) were 2/4 and 2.5 mm, respectively. By contrast, CsA-treated C57BL/6, DBA/2NCr, and C3H/HeJCr mice were comparatively resistant to MusPV1-induced papillomas. None of the virally inoculated C57BL/6 (Figure 1E) and DBA/2NCr mice (Figure S1B) developed papillomas (0/8 and 0/4, respectively, both 0%), and only one C3H/HeJCr mouse (Figure 1F) developed a lesion (1/8; 12.5%), and its length was only 2 mm.

The papillomas that did develop in the tested strains were attributable to the combination of CsA treatment and MusPV1 infection, as littermates inoculated with the same amount of MusPV1 virions in the absence of CsA or mock inoculation with CsA treatment did not result in papilloma formation (Figure 1G, 1H; SENCAR shown as representative strain). The lesions that developed were histologically verified to be papillomas with numerous koilocytes in the epithelium (Figure 1I; SENCAR shown as representative), and extracts of the lesions contained infectious MusPV1 virions that induced papillomas, in athymic NCr nude mice, whose morphology was identical to those previously reported for MusPV1 in this immunologically impaired strain (Figure 1J) [9].

Papillomas in the CsA-treated/MusPV1-inoculated immunocompetent strains grew progressively and did not regress during the period of CsA administration. However, after cessation of CsA administration, the lesions completely regressed within several weeks (Figure 1K and 1L; same SENCAR mouse at a 6-week interval). The length of time to regression depended upon the size of the lesion, with smaller ones tending to regress sooner than larger ones (Figure S1C). Therefore, the immunosuppressive phenotype resulting from CsA treatment was required for maintenance of the papillomas, in addition to their induction.

In a rabbit model, persistent PV genome expression was reported at mucosal sites of infection following lesion-regression [16]. Subsequent immunosuppression led to an increase in viral copy numbers and even to reappearance of small lesions, consistent with reactivation of latent infection [17]. To investigate latency of MusPV1, all mouse strains, except for DBA/2NCr and 129S6/SvEv, that had been inoculated with 6×10^10^ virions per animal, were subjected to CsA administration 4 months later for a period of 4 weeks (corresponding to 5 months post-infection). After this period, papilloma outgrowth was not observed in any of the animals (data not shown). MusPV1 E1∧E4 spliced transcripts, a marker for PV infection [9], [18], [19], and viral genomes were undetectable in the skin tissues taken from the inoculation sites (Figure S2), suggesting that MusPV1 infection in cutaneous tissues was efficiently cleared by a fully functioning murine immune system prior to immunosuppression and therefore do not persist long-term in a latent state in immunocompetent mice.

When susceptibility to MusPV1-induced papillomas was considered in the context of the H-2 haplotype of the mice, surprisingly, there was little correlation (Table 1). For example, although C57BL/6 and 129S6/SvEv are both H2^b^, the former strain was resistant to MusPV1-induced papilloma formation, while the latter strain had intermediate susceptibility. In addition, DBA/2NCr was resistant, while BALB/cAnNCr had intermediate sensitivity, although both strains are H2^d^. By contrast, we noted some correlation between the observed susceptibility to MusPV1-induced papillomas and their previously reported susceptibility to chemical carcinogenesis (Table 1) [10], suggesting that common mechanisms may, in part, control the relative susceptibility to MusPV1-induced papilloma formation and to chemical carcinogens. However, this correlation is based on different published studies on the strain-specific susceptibility to chemical carcinogenesis [summarized in 10] and is not complete, as DBA/2NCr is reported to be susceptible to chemical carcinogenesis, but MusPV1 inoculation did not produce papillomas, and the sensitivity of 129S6/SvEv to MusPV1 may be less than its reported sensitivity to chemical carcinogenesis. Further experimentation is required to validate the partial correlation between strain-specific susceptibility to papillomatosis and chemical carcinogens.

Next, we investigated the impact of varying doses of MusPV1 virions on representatives of the most sensitive and the most resistant strains, SENCAR and C57BL/6, respectively. Serial titrations of the inocula revealed that 2–3 weeks after infection with very high doses of 1×10^12^ MusPV1 virions per animal, the majority of the SENCAR mice developed small lesions at the site of infection (covering about 1 cm of the length of the tails) (Table S1), even in the absence of immunosuppression. Sporadic papilloma outgrowth was observed after infection with 1×10^11^ MusPV1 virions. All lesions spontaneously regressed within 1–2 weeks after formation (corresponding to 3–5 weeks post-infection). Consistently, lesions did not arise with lower amounts of inocula ranging from 1×10^10^ to 1×10^8^ virions. The transient papillomas in the immunocompetent SENCAR mice obtained after inoculation with 1×10^12^ MusPV1 virions were morphologically and histologically similar to papillomas that formed under CsA immunosuppression and to those previously reported [9] (Figure S3A and S3B) and contained low, but detectable MusPV1 E1∧E4 spliced transcripts, a marker for infection (Figure S3C, lane designated M). In the epithelium of these papillomas, expression of the major capsid protein L1 was demonstrated by immunofluorescent microscopy (Figure S3D, E). Cell extracts derived from these papillomas contained infectious MusPV1 virions that were able to induce papilloma formation on the tails of athymic nude NCr mice after experimental transmission (Figure S3F), indicating that MusPV1 could be propagated in immunocompetent SENCAR mice. For C57BL/6 mice no tumor formation was observed, even at very high doses of 1×10^12^ MusPV1 virions, further supporting the higher resistance of this particular strain to MusPV1 pathogenesis (Figure S3G).

### Strain-specific papilloma outgrowth in CsA-treated/MusPV1-infected mice correlates with viral gene expression

To investigate whether qualitative and/or quantitative differences in infection parameters were responsible for the observed variation in papilloma formation between strains, the relative level of MusPV1 E1∧E4 spliced transcripts were determined in skin tissues taken 4 weeks after infection with 4.2×10^10^ MusPV1 virions (corresponding to 5 weeks of CsA administration) from the virally inoculated sites of all strains, except for DBA/2NCr and 129S6/SvEv (Figure 1M and Table 1). Skin necropsies were taken from all animals within the remaining six tested strains, including those mice that lacked visible lesions. To ensure comparability, specimens of approximately the same size were processed, and the levels of the viral transcripts were adjusted to level of endogenous beta-actin transcripts from the same sample (Figure 1M, the lanes designated CM). The resulting ratios corresponded well to the macroscopic appearance of the lesions. High levels of MusPV1 E1∧E4 copies relative to beta-actin levels were detected in tissues of CsA-treated/MusPV1-infected SENCAR and FVB/NCr mice. In CsA-treated/MusPV1-infected BALB/cAnNCr and A/JCr mice, the lower levels of E1∧E4/beta-actin reflected the less pronounced and smaller lesions observed in these intermediately susceptible strains. Consistent with the lack of visible papillomas, even lower levels of MusPV1 E1∧E4 spliced transcripts were detected in infected C57BL/6 and C3H/HeJCr mice treated with CsA. In tissues taken from MusPV1-infected littermates that had not received CsA, the levels of E1∧E4 spliced transcripts per copy beta-actin were low, but detectable, in all strains (Figure 1M, lanes designated M), but were not detected in mock-infected mice (Figure 1M, lanes designated 0). Thus MusPV1 established persistent asymptomatic infections in all of the immunocompetent mouse strains.

Immunofluorescent microscopy was used to examine the relative expression of the major capsid protein L1 in these tissues. SENCAR was used as the high susceptibility strain, BALB/cAnNCr as the intermediate one, and C57BL/6 as the resistant one. Given the correlation between the susceptibility of these strains to papilloma formation and their relative level of E1∧E4 spliced transcripts, it was anticipated that a similar correlation would be seen for L1 expression. However, there was abundant L1 staining in the epithelium of all CsA-treated/MusPV1-infected animals of all three strains (Figure 1N–P), including the resistant C57BL/6 mice, which did not develop papillomas. Similar to previous reports in athymic NCr nude mice [9], punctate L1 expression was found in the cytoplasm of keratinocytes in the basal and lower spinous layers of the epithelium, while nuclear L1 expression was confined to the upper spinous and granular layers, suggesting active virion production in these more differentiated epithelial layers. Additionally, some L1 positive squames that presumably enclose matured virions prior to shedding were found in these tissues. L1 expression was restricted to the epithelial compartment, although seemingly positive staining could occasionally be observed below the basement membrane due to the (trans-)sectioning of the papilloma's deregulated architecture. L1 staining was not detected at sites of lesion regression following cessation of CsA treatment (data not shown) or in the skin of untreated MusPV1-infected mice at this time point, regardless of the strain (Figure 1Q–S). It remains to be determined whether CsA treatment deregulates L1 protein expression to a greater degree than that of the E1∧E4 spliced transcripts, or if a difference in sensitivities between the two assays might explain the disparate results. Interestingly, L1 was readily detected in infected sites on day 14 after inoculation in untreated mice of all three strains, raising the possibility that adaptive immune responses arising between two and four weeks may regulate late gene expression and thereby virion production (data not shown).

### MusPV1 infection recruits CD4^+^ and CD8^+^ T cells to the site of infection

**Figure 2:**
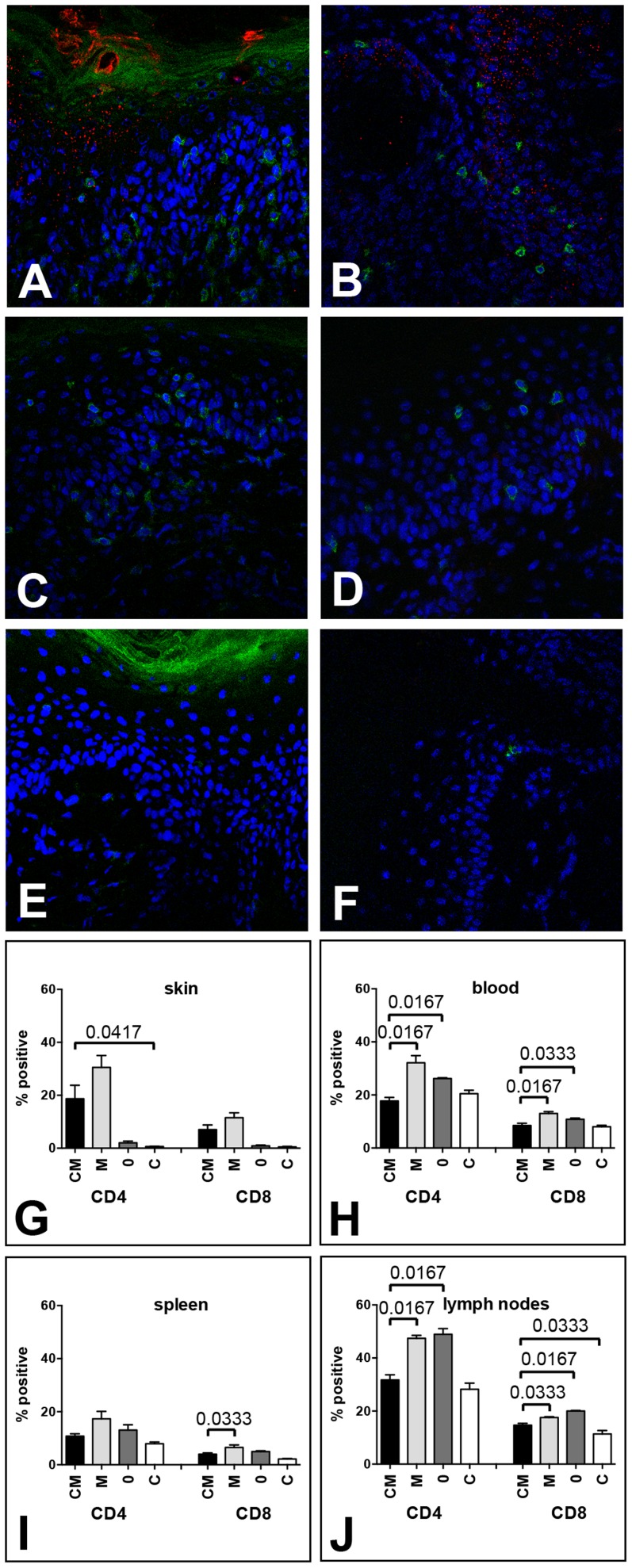
MusPV1 infection recruits CD4^+^ and CD8^+^ T cells to the site of infection in Cr:ORL SENCAR mice. Skin tissues were harvested 4 weeks after infection with 4.2×10^10^ MusPV1 virions and analyzed by immunofluorescent staining. MusPV1 L1 expression (red, detected with an Alexa Fluor 594-labeled secondary antibody) was restricted to papillomatous tissues from (A, B) CsA-treated/MusPV1-infected Cr:ORL SENCAR mice, and absent in non-papillomatous tissues of (C, D) untreated, MusPV1-infected and (E, F) mock-infected littermates. Co-detection of either CD4^+^ T cells (left panel; A, C, E) or CD8^+^ T cells (right panel; B, D, F) was performed with anti-mouse CD4 or anti-mouse CD8 antibodies conjugated with Alexa Fluor 488 (green). Epidermal and dermal infiltrates consisting of numerous CD4^+^ T cells were found in the skin tissues from (A) CsA-treated/MusPV1-infected and (C) untreated, MusPV1-infected Cr:ORL SENCAR mice. In contrast, in the skin of (E) mock-infected controls CD4^+^ T cells were sparse. Similarly, many CD8^+^ T cells were found in (B) CsA-treated/MusPV1-infected and (D) MusPV1-infected Cr:ORL SENCAR mice, but cells were localized predominantly in the epithelium. In (F) mock-infected controls only a few intraepithelial CD8^+^ T cells were observed. Quantification of CD4^+^ T cells (left side) and CD8^+^ T cells (right side) by flow cytometry analyses in (G) skin tissues, (H) blood, (I) spleen and (J) draining lymph nodes harvested 4 weeks post-infection from CsA-treated/MusPV1-infected (**CM**), MusPV1-infected (**M**), mock-infected (**0**) and CsA-treated/mock-infected mice (**C**) confirmed the strong increase of both T cell populations at the local sites of infection, even in the presence of CsA.

The recruitment of T cells to the infected tissue was evaluated in the SENCAR mice, which developed large papillomas with CsA treatment. Tissue taken 4 weeks after inoculation with 4.2×10^10^ MusPV1 virions (corresponding to 5 weeks of CsA administration) was analyzed for the presence of CD4^+^ and CD8^+^ T cells. By immunofluorescent staining (IFS), higher numbers of both T cell subsets were present in the MusPV1-infected tissue, independent of whether the mice had been treated with CsA, although L1 expression was only detected in the infected CsA-treated mice (Figure 2A and 2B). Many CD4^+^ T cells infiltrated both the epithelium and the underlying dermis in the papillomatous tissue of the CsA-treated mice and the non-papillomatous tissue of the untreated mice (Figure 2A and 2C), in contrast to the limited number of CD4^+^ T cells in the mock-infected control skin (Figure 2E). Similarly, numerous CD8^+^ lymphocytes were found in the papillomas of CsA-treated/MusPV1-infected mice and in the macroscopically unchanged MusPV1-infected skin of untreated animals (Figure 2B and 2D). However, the CD8^+^ T cells were localized predominantly in the epithelium and were sparse in the dermis. Only a few widely spaced intraepithelial CD8^+^ T cells were found in the mock-infected control skin (Figure 2F).

Consistent with these findings, quantification of CD4^+^ and CD8^+^ T cell numbers in the infected skin tissues (Figure 2G) by flow cytometry confirmed that MusPV1 induced strong recruitment of the T cells even in CsA-treated mice. Viral infection without CsA resulted in a 15.3- and 12.8-fold increase in CD4^+^ and CD8^+^ T cell numbers, respectively, compared to mock-infected littermates. Similarly, viral infection with CsA treatment resulted in an increase in CD4^+^ and CD8^+^ T cells that was, respectively, 9.3- and 7.8-fold higher when compared with mock-infected mice not treated with CsA, or was, respectively, 31.1- and 15-fold higher, compared to mock-infected/CsA-treated controls. Thus, MusPV1 infection efficiently recruits both T cell subsets to the site of infection, even in the presence of CsA.

However, when the same mice were analyzed for their CD4^+^ and CD8^+^ T cell levels in blood (Figure 2H), spleen (Figure 2I), and draining lymph nodes (Figure 2J), the impact of MusPV1 infection was much less pronounced. Although some MusPV1-dependent differences seen in the CD4^+^ or CD8^+^ T cells in these systemic compartments were statistically significant, there was less than a two-fold difference for each comparison.

### In SENCAR mice, depletion of CD3^+^ T cells permits papilloma formation

To investigate the role of T cells as key effectors responsible for controlling MusPV1 infection and/or papilloma formation, we studied the consequences of MusPV1 infection following antibody-induced depletion of specific T cell populations in two mouse strains: SENCAR, which had been found to be highly sensitive to papilloma formation following CsA treatment, and C57BL/6, which had been found to be resistant. The SENCAR results are presented in this section and the C57BL/6 results in the next section.

**Figure 3:**
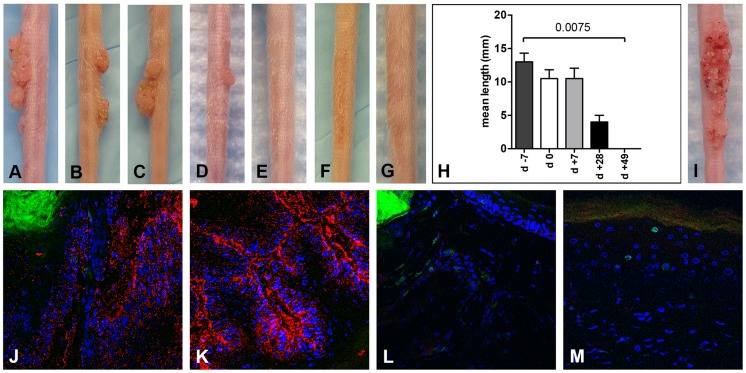
CD3^+^ T cell depletion allows papilloma formation in Cr:ORL SENCAR mice. Infection of Cr:ORL SENCAR mice was performed on day 0 using 7.3×10^10^ MusPV1 virions and depletion of CD3^+^ T cells achieved by administration of anti-murine CD3 monoclonal antibodies for a total of 7 weeks. Efficient papilloma formation was observed when CD3^+^ immunodepletion was started (A) 7 days prior to, (B) on the day of, and (C) 7 days after infection. Efficiency was markedly reduced when depletion was started (D) 28 days post-infection, and no papilloma outgrowth was observed when depletion was started (E) 49 days after infection. (F) Isotype-depleted MusPV1-infected control animals and (G) MusPV1-infected control mice did not develop papillomas. (H) Comparison of tail lesions in these animals after 7 weeks of T cell depletion showed the differences in mean lesion lengths, given in mm, between the different experimental groups. Data represent the mean ± SEM of five mice/group from a representative experiment. (I) MusPV1 virions that had been extracted from lesions of CD3^+^ T cell depleted/MusPV1-infected Cr:ORL SENCAR mice gave rise to papillomas on the tail of an athymic NCr nude mouse after experimental transmission. (J–K) Immunofluorescent staining revealed exceptionally large amounts of MusPV1 L1 protein (red, detected with an Alexa Fluor 594-labeled secondary antibody) in the papillomatous lesions taken from MusPV1-infected Cr:ORL SENCAR mice after 7 weeks of CD3^+^ T cell depletion (day −7 group shown as representative). (L–M) In contrast, L1 expression was absent in skin tissues taken from isotype-depleted controls. Co-stainings of (J, L) CD4^+^ T cells or (K, M) CD8^+^ T cells in the tissues were performed using Alexa Fluor 488-labeled anti-CD4 or anti-CD8 antibodies (green), respectively, and confirmed the absence of T cells in the depleted animals. Infection of all animals was performed on day 0 using 7.3×10^10^ MusPV1 virions.

In the first experiment, the CD3^+^ T cell population, which includes most T cell subsets, was specifically removed from the SENCAR mice by systemic administration of a monoclonal antibody (mAb) recognizing murine CD3. Depletion was started at various time points, from one week prior to viral infection (day −7) to 7 weeks (day +49) after infection, and it was maintained for seven weeks for all groups. The mice were infected on day 0 with 7.3×10^10^ MusPV1 virions, and the number and size of lesions were determined for each group after 7 weeks of immunodepletion (Figure 3A–H). Depletion starting 1 week prior to infection (day −7; Figure 3A) resulted in the development of large papillomas (mean length = 13 mm) in all 5 mice in this group. When depletion was started on the day of infection (day 0; Figure 3B) or 1 week after infection (day +7; Figure 3C), most mice developed papillomas (4/5 in both groups), but their size was somewhat smaller (mean length = 10.5 mm for both groups). The efficiency of papilloma formation was markedly reduced (2/5) when depletion was started 1 month post-infection (day +28; mean length = 4 mm; Figure 3D). No papillomas were seen when depletion was started 7 weeks after infection (day +49; 0/5; Figure 3E), suggesting that MusPV1 infection is effectively cleared prior to this time. MusPV1 inoculation after administration of an isotype control (Figure 3F) or without mAb addition (Figure 3G) did not induce papilloma formation. The papillomas in the CD3-depleted mice were histologically verified (data not shown), and virions extracted from these lesions were able to cause papillomas in athymic NCr nude mice (Figure 3I), thus demonstrating that depletion of CD3^+^ T cells enables the complete viral lifecycle.

Throughout the papillomatous tissues taken after 7 weeks of depletion (corresponding to 6 weeks post-infection) from the CD3-depleted mice (Figure 3J and 3K; representative of group day −7 shown), exceptionally large amounts of MusPV1 L1 protein were detected by IFS, and CD4^+^ (Figure 3J) and CD8^+^ (Figure 3K) T cells were absent, as expected, since these T cells are CD3^+^. In contrast, skin tissues taken from isotype-administered controls (Figure 3L and 3M) lacked MusPV1 L1 protein, and CD4^+^ (Figure 3L) and CD8^+^ (Figure 3M) T lymphocytes were readily detectable. No L1 protein and only isolated T cells were observed in mock-infected controls (data not shown).

Taken together, these findings indicate that, in SENCAR mice, T cells are obligatory for controlling MusPV1 infection and associated papillomatosis. T cell-mediated responses either clear MusPV1 infection within 7 weeks post-inoculation or control it by a mechanism that does not permit reactivation after their removal.

### Depletion of CD4^+^ or CD8^+^ T cells in SENCAR mice allows papilloma outgrowth

**Figure 4:**
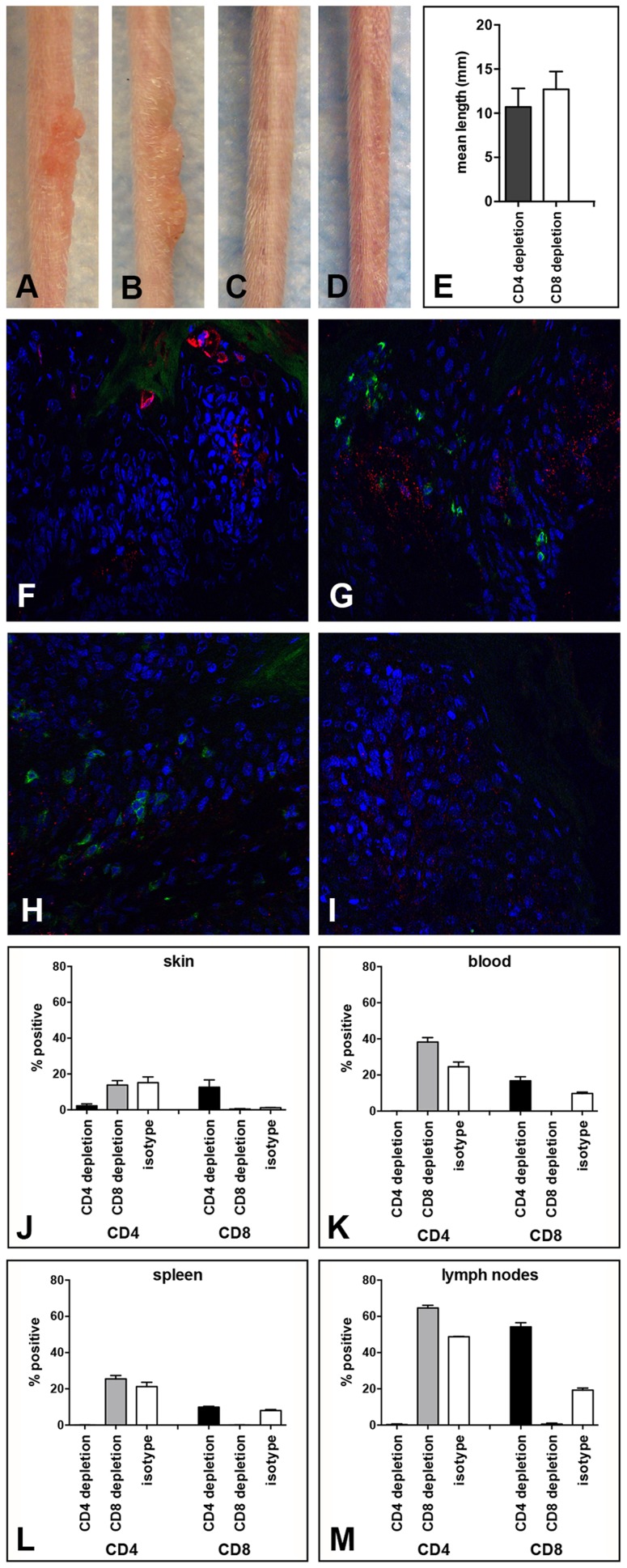
Papilloma formation can be observed in CD4- or CD8-depleted Cr:ORL SENCAR mice. Antibody-mediated depletion of (A) CD4^+^ T cells allows papilloma formation in Cr:ORL SENCAR mice after 7 weeks of immunodepletion (6 weeks post-infection). (B) Similarly, papilloma formation was observed in CD8-depleted littermates at the same time point. (C) Isotype-depleted and (D) MusPV1-infected controls did not develop papillomas. All mice were infected with 5.1×10^9^ MusPV1 virions. (E) Comparison of tail lesions of CD4- and CD8-depleted animals at this time point showed comparable mean lesion lengths, in mm, in these animals. Data represent the mean ± SEM of nine mice/group from a representative experiment. Immunofluorescent staining of skin tissues from (F,G) CD4-depleted or (H, I) CD8-depleted mice demonstrated abundant expression of MusPV1 L1 proteins (red, detected with an Alexa Fluor 594-labeled secondary antibody) in these tissues. Co-stainings using an Alexa Fluor 488-labeled anti-CD4 (F, H) or an Alexa Fluor 488-labeled anti-CD8 (G, I) antibody (green) confirmed the absence of the targeted T cell subpopulation in the tissues. There was no loss in the infiltration by the non-depleted subset. Quantification of CD4^+^ (left side) and CD8^+^ (right side) T lymphocytes in (J) skin, (K) blood, (L) spleen and (M) draining lymph nodes of CD4-, CD8- and isotype-depleted MusPV1-infected Cr:ORL SENCAR mice by flow cytometry analyses was performed after 5 weeks of immunodepletion (corresponding to 4 weeks post-infection) and demonstrated the efficient and specific depletion of the targeted subpopulation in each compartment.

We next determined whether depletion of either the CD4^+^ or the CD8^+^ T cell population alone, by administration of anti-CD4 or anti-CD8 mAbs, respectively, would be sufficient to induce sensitivity to MusPV1-induced papillomas in the SENCAR mice. The immunodepletion was started one week prior to infection with 5.1×10^9^ MusPV1 virions, and the depleted state was monitored in the animals' blood prior to infection (day −1) and every 2 weeks by flow cytometric analysis (Figure S4). At six weeks post-infection, the majority of the CD4-depleted SENCAR mice had developed papillomas (7/9; 78%) (Figure 4A). Similarly, there were papillomas in 7/9 (78%) of the CD8-depleted mice (Figure 4B) at this time point. MusPV1-infected controls with (Figure 4C) and without (Figure 4D) isotype depletion did not develop lesions. The mean length of the lesions was similar in the CD4-depleted and CD8-depleted groups, being 10.7 mm vs. 12.7 mm, respectively (Figure 4E). The fact that removal of either subset allowed papilloma outgrowth suggests that cooperation between CD4^+^ and CD8^+^ T cells is required for effective control of infection and lesion development in SENCAR mice.

As expected, MusPV1 L1 protein was present in skin tissues taken at this time point from both CD4- (Figure 4F and 4G) and CD8-depleted (Figure 4H and 4I) animals. The L1 staining pattern and the intensity were similar to the pattern observed in CsA-treated/MusPV1-infected and athymic NCr nude mice [9], with punctate, cytoplasmic L1 expression in the lower epithelium and nuclear L1 positivity in the upper epithelial layers. Compared to the results obtained after CD3^+^ T cell depletion, L1 expression seemed less pronounced, but this difference may be due to the lower amount of virus used for the initial inoculation.

Depletion of the targeted T cell subset at the site of infection was associated with the apparent absence of the depleted subset but with no loss in the infiltration by the non-depleted subset, indicating the neither subset was needed for recruitment of the other (Figure 4F–I). After 5 weeks of depletion (corresponding to 4 weeks post-infection), when the growth of papillomas was clearly visible, lymphocytes from each group (4 mice/group) were isolated from the site of infection (Figure 4J), and the blood (Figure 4K), spleen (Figure 4L) and draining lymph nodes (Figure 4M) for analysis by flow cytometry. The results in the skin confirmed the validity of the microscopy results, and demonstrated the efficient and specific depletion of the targeted subpopulation in each compartment at this time point.

### CD3^+^ or combined CD4^+^/CD8^+^ T cell removal, but not single CD4^+^ or CD8^+^ T cell depletion results in papilloma formation in C57BL/6 mice

**Figure 5:**
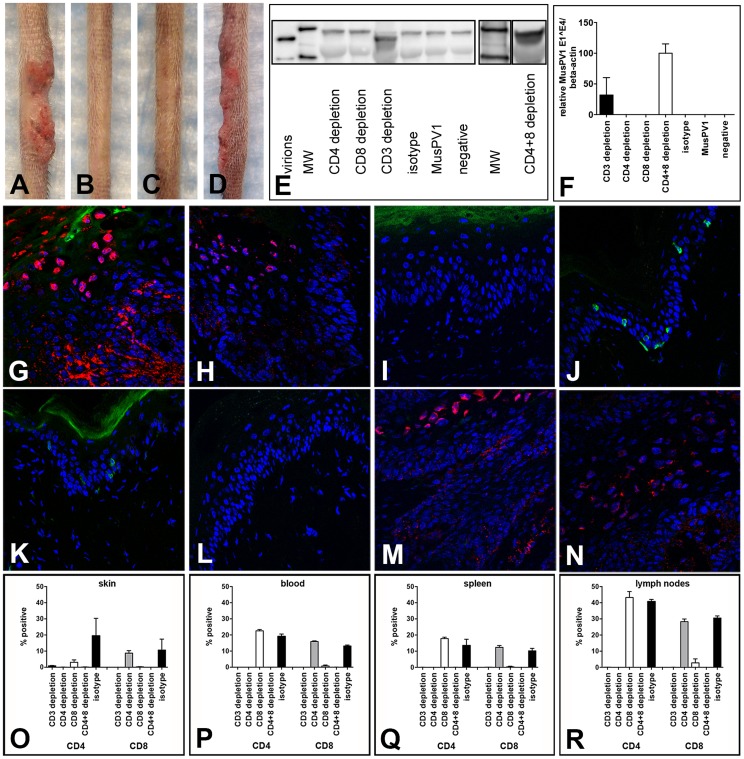
CD3^+^, but not single CD4^+^ or single CD8^+^ T cell depletion allows papilloma formation in C57BL/6 mice. Wild-type C57BL/6 mice were infected with 5.3×10^10^ MusPV1 virions, and the depleted state maintained for a total of 7 weeks. After this period papillomas developed in (A) CD3-depleted (n = 14), but not in (B) single CD4-depleted (n = 5) and (C) single CD8-depleted (n = 5) C57BL/6 mice. (D) Combined CD4+8 depletion allowed papilloma outgrowth in C57BL/6 mice (n = 10). (E) MusPV1 L1 protein at the expected size of 55–60 kD was found by Western Blot in crude skin tissue lysates prepared from CD3 and combined CD4+8-depleted C57BL/6 mice, but was absent in tissue lysates from single CD4-depleted or single CD8-depleted animals. L1 protein was also undetectable in MusPV1-infected, non-depleted and mock-infected littermates. One representative per group is shown. The molecular weight marker (MW) is depicted on the left side and 60 kD and 50 kD markers are visible; purified MusPV1 virions served as controls. (F) MusPV1-specific E1∧E4 spliced transcripts relative to endogenous beta-actin were detected at high levels in skin tissues harvested at 6 weeks post-infection (corresponding to 7 weeks of depletion) from CD3 and combined CD4+8-depleted C57BL/6 mice. E1∧E4 spliced transcripts were undetectable in tissues from CD4-depleted, CD8-depleted animals, and appropriate controls. Immunofluorescent staining showed the presence of MusPV1 L1 protein (red, visualized using an Alexa Fluor 594-labeled secondary antibody) in skin tissues harvested after the 7 week depletion period from (G,H) CD3-depleted and (M,N) combined CD4+8-depleted C57BL/6 mice. Consistent with the lack of E1∧E4 spliced transcripts or L1 protein expression in crude lysates, L1 protein was undetectable in tissues from (I,J) single CD4-depleted and (K,L) single CD8-depleted. C57BL/6 mice. Co-staining of (G, I, K, M) CD4^+^ T cells with a directly Alexa Fluor 488-labeled anti-CD4 antibody (green) or co-staining of (H, J, L, N) CD8^+^ T cells confirmed the efficient and specific immunodepletion in these tissues. Flow cytometry analyses of CD4^+^ (left side) and CD8^+^ (right side) T cells in (O) skin tissues, (P) blood, (Q) spleen and (R) draining lymph nodes of T cell depleted C57BL/6NCr mice further corroborated maintenance of the depleted state in each compartment.

T cell depletion experiments analogous to those described for the SENCAR mice were performed in C57BL/6 mice. MAb-induced depletion of CD3^+^ T cells was initiated one week prior to infection with 5.3×10^10^ MusPV1 virions, and the depleted state was monitored in the animals' blood one day prior to infection and every second week (Figure S5). In parallel, mAb-induced depletion was initiated, for the same length of time, for the CD4^+^ T cells, the CD8^+^ T cells, and both the CD4^+^ and the CD8^+^ T cells (CD4+8 depletion), and the depletion monitored similarly (Figure S5). After 7 weeks of depletion (corresponding to 6 weeks post-infection), all of the CD3-depleted animals (14/14) inoculated with MusPV1 had developed papillomas (100%) (Figure 5A), similar to the results observed in the SENCAR mice. This finding was somewhat unexpected, given that continuous CsA treatment of the C57BL/6 mice had not led to the development of MusPV1-induced papillomas (Figure 1E). On the other hand, the mice with depletion of only CD4^+^ T cells (0/5) (Figure 5B) or of only CD8^+^ T cells (0/5) (Figure 5C) did not develop papillomas, which is in contrast to the results with the SENCAR mice under the same conditions. However, combined CD4+8 depletion in the C57BL/6 mice did lead to MusPV1-induced papillomas (10/10) (Figure 5D).

When crude skin extracts taken from the mice 6 weeks after infection were evaluated by western blotting for the presence of L1 protein, it was only detected in the two groups that developed papillomas, CD3 depletion and the combined CD4+8 depletion (Figure 5E).

Given that the positive results with C57BL/6 mice were seen with CD3 depletion or combined CD4+8 depletion induced by mAb, further analysis was focused on depletion induced by these mAb. At 6 weeks after virus inoculation, high levels of MusPV1 E1∧E4 spliced transcripts, as a measure of persistent infection, were found in infected skin tissues of CD3- and combined CD4+8-depleted animals (Figure 5F), the two depletion conditions that resulted in papillomas. In contrast, no E1∧E4 spliced transcripts were detected in CD4- or in CD8-depleted mice, demonstrating the effective control of MusPV1 infection in these animals.

When the expression of L1 protein at the inoculated skin sites was examined by IFS, the results corroborated the results observed with the E1∧E4 spliced transcripts. L1 was abundantly expressed in the cytoplasm and nuclei of infected keratinocytes in the characteristic pattern of MusPV1 in mice with CD3 depletion and CD4+8 depletion, but not with CD4 depletion or CD8 depletion (Figure 5G–5N). As expected, no CD4^+^ cells were seen in skin sites from CD3-depleted, CD4+8-depleted, or CD4-depleted mice, although they were detected in CD8-depleted mice (Figure 5G, 5M, 5I and 5K). Conversely, no CD8^+^ cells were seen in skin sites from CD3-depleted, CD4+8-depleted, or CD8-depleted mice, although they were detected in CD4-depleted mice (Figure 5H, 5N, 5L and 5J). Quantification of T cell infiltrates in the infected skin sites (Figure 5O) and the blood (Figure 5P), spleen (Figure 5Q), and draining lymph nodes (Figure 5R) by flow cytometric analyses further confirmed the efficiency and specificity of the T cell depletion.

### C57BL/6 mice constitutively deficient for CD4 or CD8 do not develop papillomas after MusPV1 infection

**Figure 6:**
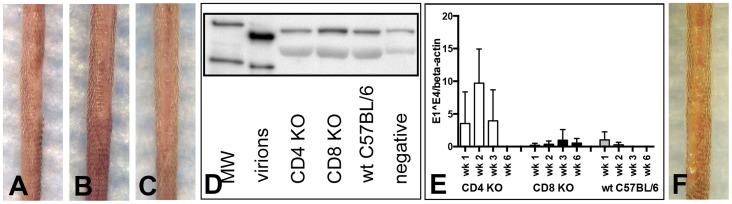
CD4- and CD8-deficient C57BL/6 mice differ in MusPV1 gene transcription early after infecton. (A) CD4-deficient (n = 5), (B) CD8-deficient (n = 5), and (C) wild-type C57BL/6 mice did not develop papillomas within 3 months after infection with 9.4×10^10^ MusPV1 virions. (D) Western Blot analysis for the presence of MusPV1 L1 protein in crude skin tissue extracts taken from MusPV1-infected CD4-deficient, CD8-deficient, and wild-type C57BL/6 mice as well as from mock-infected controls verified the lack of MusPV1 L1 protein in these tissues. One representative per group is shown. The molecular weight marker (MW) is depicted on the left side and 60 kD and 50 kD markers are visible; purified MusPV1 virions served as controls. (E) High levels of MusPV1 E1∧E4 spliced transcripts relative to endogenous beta-actin were determined in skin tissues from CD4-deficient C57BL/6 mice during the first 3 weeks and became undetectable within 6 weeks after infection. Very low, but detectable levels of MusPV1 E1∧E4 transcripts per beta-actin were observed in CD8-deficient C57BL/6 mice. MusPV1 E1∧E4 levels were very low up to 2 weeks post-infection in wild-type C57BL/6 mice and became undetectable after week 3 post-infection. Data represent the mean ± SD of five mice/group from a representative experiment. (F) CD1d-deficient C57BL/6 mice (n = 5) did not develop papillomas within 3.5 months after infection with 6.8×10^10^ MusPV1 virions.

To address whether C57BL/6 mice genetically engineered to be knock-outs (KO) for CD4^+^ or CD8^+^ T cells might be susceptible to papilloma induction by MusPV1, these KO mice were inoculated with 9.4×10^10^ MusPV1 virions. However, as had been true of the mice with mAb-induced depletion of these individual T cell subsets, the CD4 KO mice (0/5) (Figure 6A) and the CD8 KO mice (0/5) (Figure 6B) were resistant to papilloma formation when observed for 3 months, as were wild-type (wt) C57BL/6 controls (0/5) (Figure 6C), and did not produce detectable levels of L1 protein in crude skin extracts at this time point (Figure 6D).

However, analysis of MusPV1 E1∧E4 spliced transcripts, as a measure of infection, at earlier time points (Figure 6E) revealed substantially higher numbers of MusPV1 E1∧E4 spliced transcripts relative to beta-actin in CD4 KO mice in the first three weeks post-infection, compared to wt C57BL/6 and CD8 KO mice. The relative MusPV1 E1∧E4 levels in the CD4 KO mice peaked at week 2 post-infection, showing 36-fold and 27-fold higher values than in wt and CD8-deficient C57BL/6 mice, respectively, gradually decreased thereafter and became undetectable at week 6 post infection. Very low numbers of MusPV1 E1∧E4 transcripts could be detected within the first 2 week after infection in wt C57BL/6 mice and were undetectable 3 weeks after infection. In contrast the levels of MusPV1 E1∧E4 transcripts in the CD8 KO mice remained uniformly very low, but detectable, throughout the experiment, suggesting that CD4^+^ T cells are specifically involved in early, presumably innate, immune responses that initially control viral gene expression.

CD1d-deficient C57BL/6 mice, which selectively lack the natural killer (NK)-T cell population, also failed to form papillomas after inoculation with 6.8×10^10^ MusPV1 virions and a 3.5 month observation period (Figure 6F), indicating that ablation of this subset by itself is not sufficient to permit papilloma formation, in contrast to the crucial role of T cells.

## Discussion

In this study, we have determined that a variety of immunocompetent mouse strains are resistant to papilloma induction by MusPV1, although a limited degree of virus expression can be detected at 4 weeks after infection. However, immunosuppression induced by CsA uncovered a strain-dependent hierarchy in the degree of susceptibility to papilloma formation and virus production. Papilloma formation correlated more closely with expression of E1∧E4 spliced transcripts than with expression of the major L1 capsid protein, as clinically normal virally inoculated skin sites in the resistant C57BL/6 mouse had relatively high levels of L1, but low E1∧E4 levels. Inoculation with what we assume are super-physiological amounts of MusPV1 virions (1×10^12^ virions) seems to overcome early control of infection in the highly susceptible SENCAR strain, allowing for transient papilloma formation. In the more resistant C57BL/6 strain, papilloma outgrowth was not observed after this stringent challenge, supporting the conclusion that this strain is better able to control infection than SENCAR mice.

To date, strong circumstantial evidence supports the role of cell-mediated immunity, especially T cells, in controlling and eliminating established PV infection and neoplasia. Evidence for the pivotal role of T cells has emerged from studies of humans infected with the human immunodeficiency virus [20]. In these individuals, higher prevalence of HPV infection, especially of the anogenital tract, viral persistence, and very often the presence of multiple types, has been observed. An important role for T cells is also supported by observations that iatrogenic immunosuppressed transplant recipients have high rates of extensive viral warts, HPV-associated anogenital cancers, and non-melanoma skin cancer [3]. The current study demonstrated that treatment with CsA, whose predominant activity is against T cells and which is routinely used in humans, led to papilloma formation and persistent MusPV1 infection in most, but not all, of the murine strains tested. However, the finding that depletion of CD3^+^ T cells rendered the mice susceptible to papilloma formation and exuberant viral infection, even in C57BL/6 mice that were resistant after CsA treatment, provides direct experimental evidence for the critical importance of T cell function in the control of MusPV1 infection.

The available data with other PV systems have not defined the T cell subpopulation(s) that is the key player responsible for virus control and papilloma regression. Observational studies in humans have found that regression of anogenital warts is accompanied by a massive infiltration of CD4^+^ lymphocytes, both within the papilloma's stroma and the epithelium [21]. However, intraepithelial CD8^+^ T cells have also been associated with regression of cervical intraepithelial lesions [22], [23]. T cell infiltrates, consisting of predominantly CD4^+^ T cells but also containing CD8^+^ T cells, have been described in regressing mucosal lesions caused by BPV type 4 [24], canine oral PV [25], and rabbit oral PV [26]. In cutaneous papillomas caused by CRPV, a mostly CD8^+^ T cell infiltration of the epithelium, with very few accompanying CD4^+^ T cells, was demonstrated [27].

In our murine system, MusPV1 infection of the sensitive SENCAR mouse induced a dense CD4^+^ T cell infiltrate in the dermal and the epithelial compartment, as well as an intraepithelial one composed of CD8^+^ T cells, whether the animals developed papillomas as a result of the immunosuppressive CsA treatment or had clinically normal skin because they had not been immunosuppressed. Thus, the infiltrate was similar, independent of whether the mouse developed lesions, indicating that functional properties of the T cells, rather than epithelial trafficking, were primarily affected by CsA treatment.

The availability of many immunological reagents for the domestic mouse made it possible to critically evaluate individual T cell subsets in the control of PV infection and associated neoplastic disease. In SENCAR, depletion of either CD4^+^ or CD8^+^ T cells allowed papillomatosis. By contrast, combined depletion of CD4^+^ and CD8^+^ T cells was necessary for papilloma development in C57BL/6. These discrepant results were confirmed using genetically modified C57BL/6 mice deficient in either CD4^+^ or CD8^+^ T cells.

Clearance of virally-infected epithelial cells and regression of epithelial neoplasia have most often been attributed to CD8^+^ T cells, especially cytotoxic ones. However, in many well-characterized systems, CD4^+^ T help is necessary both for induction of primary CD8^+^ T cell responses and for their proliferation, activation, and differentiation into effector cytotoxic T lymphocytes (CTL) [28]–[30]. In the absence of CD4^+^ T help, CD8^+^ T cells often fail to acquire antiviral effector functions, including the ability to produce antiviral cytokines, such as interferon (IFN)-γ and tumor necrosis factor (TNF)-α, and cytotoxic molecules, such as perforin and granzymes. In this scenario, CD4^+^ T cells would contribute to protection indirectly, rather than directly providing a critical T cell effector function. However, the T cell subset depletion and knock-out data in C57BL/6 data make it clear that protective antiviral CD8^+^ T cell responses to a PV infection can develop in the absence of CD4^+^ T help, and that CD4^+^ T cell-dependent effector functions can control PV infection in the absence of CD8^+^ T cell functions.

There is an increasing body of evidence that CD4^+^ T helper-independent CD8^+^ CTL responses can be elicited by some pathogens, such as ectromelia virus [31], influenza virus [32], lymphocytic choriomeningitis virus, dengue virus [33], and Listeria monocytogenes [34]. However, CD4^+^ T cells were required for efficient local recruitment of herpes simplex virus-specific CD8^+^ T cells to the vaginal epithelium in a murine model of herpes virus infection [35]. It was therefore unexpected that, in both C57BL/6 and SENCAR mice, CD8^+^ T cells infiltrated the skin sites inoculated with MusPV1 whether or not the mice contained CD4^+^ T cells, and vice versa. The discrepant conclusions between our study and the murine herpes virus infection report [35] may be attributable to substantial differences in the two experimental systems, most notably the adoptive transfer of transgenic CD8^+^ T cells in the herpes virus study and the *de novo* generation of the T cells by *in situ* virus infection in the current study.

Various mechanisms for CD8^+^ T cell activation in the absence of CD4^+^ T cell help have been proposed, such as direct signaling through CD40 present on antigen-presenting cells [36], [37], up-regulation of CD40L on dendritic cells to enhance CD8^+^ T cell responses via direct engagement of CD40 on activated CD8^+^ T cells [38], and NK cell-derived IFN-γ [39]. These bypass mechanisms may also be active in C57BL/6 mice and explain the observed CD4-independent suppression of MusPV1-induced papillomatosis.

CD4^+^ T cells could independently control PV infection by either a cytokine-mediated mechanism or by direct cytotoxicity [40]. Several recent studies have reported that cytolytic CD4^+^ T lymphocytes, which may represent a new CD4^+^ lineage, can contribute to the control of certain viral infections [40]–[42]. In C57BL/6 mice, lymphochoriomeningitis virus-specific CD4^+^ T cells are capable of *in vivo* cytolytic killing of peptide pulsed MHC II-positive lymphocytes [43]. However, none of these studies have demonstrated complete protection from productive infection and disease in the absence of CD8^+^ T cells.

Cytolysis by CD4^+^ T cells would likely require a direct interaction of the T cells with MHC class II molecules on the infected keratinocytes (the only cell type that is normally infected by PVs). We did detect CD4^+^ T cells within the epithelium at sites of infection, in addition to being present in the dermis, and keratinocytes can express class II molecules under certain inflammatory conditions, e.g. in response to (IFN)-γ [44]. However, we failed to detect MHC class II expression on keratinocytes at sites of infection, both when the infection was being controlled by the immune system and when it was not (unpublished data). Therefore we currently favor the hypotheses that CD4^+^ T cells are activated by cross-presentation of viral antigens by professional antigen presenting cells, and PV infection is controlled via soluble factors produced by the CD4^+^ T cells.

Although the details of immune recognition of MusPV1 remain to be determined, it seems likely that T cells control MusPV1 infection by both innate and adaptive immune mechanisms. The timing of papilloma regression after suspending CsA treatment and of spontaneous regression after high dose challenge in the SENCAR mice is consistent with induction of an antigen-specific adaptive response. However, the early control viral gene transcription in C57BL/6 mice at week 1–2, that is maintained in the CD8 KO mice but lost in the CD4 KO mice (Figure 6E), strongly suggests that CD4-mediated innate immune responses also plays a role in controlling infection.

Our findings raise several additional interesting issues. One is what may account for the different requirements for protection in C57BL/6 and SENCAR by CD4^+^ and CD8^+^ T cells. One possibility is that both the CD4^+^ and the CD8^+^ T cells in C57BL/6 are more potent in their ability to confer resistance than in SENCAR, making the presence of one of the T cell subsets sufficient to keep C57BL/6 resistant. This situation could arise if there were a single factor common to both T cell subsets that is more potent in C57BL/6 than in SENCAR, or if there were separate CD4^+^-specific and CD8^+^-specific factors. Alternatively, there could be a factor that, although it is extrinsic to CD4^+^ and CD8^+^ T cells, can cooperate with either subset in making a mouse resistant to papillomatosis. The observed strain-dependent difference could then be explained if the activity of this putative non-CD4/non-CD8 factor(s) is sufficiently more potent in C57BL/6 than in SENCAR that it can cooperate with CD4^+^ or CD8^+^ T cells to confer resistance in C57BL/6 but not in SENCAR.

A second issue is the partial correlation between the reported strain-dependent susceptibility to chemical-induced papillomatosis and the robustness of persistent infection and papillomatosis after CsA treatment found in this study. The observed partial correlations may be due to yet undetermined strain-specific genetic factors allowing for control of skin tumor susceptibility/resistance. The effects of immunosuppression on 7,12-dimethylbenz[a]anthracene (DMBA)/phorbol ester 12-O-tetradecanoylphorbol 13-acetate (TPA) tumorigenesis have been studied to a limited degree. In C3H/HeN, compared with wt mice, CD8 KO mice had an increased number of tumors, but numbers were decreased in CD4 KO mice [45]. In contrast to C3H/HeN, in FVB/N mice, CD8 KO mice had a decreased number of tumors [46]. It might be informative to examine the role of CD4^+^ and CD8^+^ cells in DMBA/TPA tumorigenesis in C57BL/6.

A third issue is whether the characteristics of MusPV1 infection documented herein make it an attractive model of any HPVs. At present MusPV1 infection appears to most closely resemble that of genus beta HPVs. These cutaneous HPVs have been found in immunocompetent individuals in clinically normal skin and plucked hairs where they predominantly induce asymptomatic skin infections [47], [48], similar to MusPV1 infection described herein. However, after immunosuppression, beta HPVs could be more frequently detected, tended to display higher viral loads, and most importantly induced visible lesions after immunosuppression [49]–[51]. The presence of beta-HPVs has been implicated as a causal factor in the increased risk for development of non-melanoma skin cancers in immunosuppressed transplant recipients. Thus, MusPV1 infection may provide a model to study the impact of a cutaneous PV type on the pathogenesis of non-melanoma skin cancer in an immunosuppressed and in an immunocompetent setting.

## Materials and Methods

### Animals

The following mice (H-2 haplotypes are given in parentheses) were obtained from the National Institutes of Health or The Jackson Laboratories (Bar Harbor, MN): immunocompetent outbred Cr:ORL SENCAR; immunocompetent inbred strains FVB/NCr (H2^q^), BALB/cAnNCr (H2^d^), DBA/2NCr (H2^d^), A/JCr (H2^a^), C57BL/6NCr (C57BL/6) (H2^b^), 129S6/SvEv (H2^b^), C3H/HeJCr (H2^k^); immunodeficient athymic NCr nu/nu; CD4-, CD8-, CD1d-deficient C57BL/6.

### Ethics statement

The mice, all females aged 6–10 weeks, were housed and handled in strict accordance to the National Institutes of Health guidelines for the use and care of live animals. Experimental protocols were approved by the National Cancer Institute's Animal Care and Use Committee (Permit Number LCO 027).

### Isolation of infectious MusPV1 virions

Crude extracts of papillomatous tissues were prepared as previously described [9], and MusPV1 virions purified from the extracts by Optiprep gradient centrifugation as detailed on the laboratory website (http://home.ccr.cancer.gov/Lco) [52]. In the purified preparations, MusPV1 viral copy numbers were quantified by real-time PCR [9] after liberation of encapsidated DNA from the viral capsids with proteinase K. The presence of the MusPV1 major capsid protein L1 in the purified fractions or in the crude tissue extracts was determined by Western blot using a polyclonal rabbit immune serum raised against MusPV1 L1 virus-like particles, at a dilution of 1∶1000 [9].

### *In vivo* transmission, systemic immunosuppression and T cell depletion

*In vivo* infection with purified MusPV1 virions was performed on pre-scarified skin of the animals' tails as previously published [9], [13]. The tail was chosen, as it represents a location that is highly permissive for MusPV1 infection [9]. The tail also has the advantage over the equally permissive muzzle skin, in that extensive lesional growth does not cause obvious distress to the animals. The skin on the animals' backs was not tested due to its minimal susceptibility to MusPV1 infection in athymic NCr nude mice using the same technique of inoculation [9].

For systemic immunosuppression, CsA (Sandimmune Inject., Novartis) was diluted under sterile conditions with PBS and administered subcutaneously to the animals five times per week at a dose of 75 mg/kg body weight in a volume of 0.1 ml. Treatment was started one week prior to infection and maintained for additional 4 weeks post-infection for a total of 5 weeks.

*In vivo* depletion of T cells was achieved by intraperitoneal administration of mAbs (all BioXCell): anti-mouse CD4 (clone GK1.5), anti-mouse CD8a (clone 53-6.72), anti-mouse CD3 (clone 17A2) in a dose of 0.5 mg per mouse in a volume of 0.1 ml. Rat IgG2b (clone LTF-2) or rat IgG2a (clone 2A3) mAbs were used as appropriate isotype controls. Depletion was performed on three consecutive days starting at indicated time points and the depleted state maintained by administration of mAbs twice a week for a period of 7 weeks. Depletion was verified by flow cytometry analyses as described below.

The CsA experiments (n = 4–5 animals per experimental group) were repeated twice for FVB/NCr, A/JCr and C3H/HeJCr mice, three times for BALB/cAnNCr and C57BL/6 mice, and 17 times for Cr:ORL SENCAR. The experiments using DBA/2NCr and 129S6/SvEv mice were tested only once as these haplotypes (H2^d^ and H2^b^) were already represented by BALB/cAnNCr and C57BL/6 mice, respectively. The depletion experiments in Cr:ORL SENCAR mice (n = 9 per group) and in C57BL/6 mice (n = 5 per group) were repeated twice. The experiments employing C57BL/6 KO mice (n = 5 per group) were repeated twice.

### Determination of MusPV1 E1∧E4 spliced transcripts and genome copy numbers

For detection of MusPV1 E1∧E4 spliced transcripts as a measure for initial infection, total RNA was isolated from tail skin necropsies using TRI Reagent (Molecular Research Center Inc.), treated with DNAse I (Qiagen), and reverse-transcribed into cDNA using the SuperScript III First-Strand Synthesis System (Invitrogen), following the manufacturers' instructions. Real-time PCR using primers and probe specific for MusPV1 E1∧E4 spliced transcripts was performed in an ABI PRISM 7900HT Sequence Detection System, as previously reported [9]. The results were correlated to the endogenous control, beta-actin (LifeTechnologies), in the same samples. Quantification of MusPV1 genome copy numbers were performed using MusPV1-forward and MusPV1-reverse primers and compared to defined amounts of re-ligated MusPV1 genome as standards, as described previously [9].

### Hematoxylin-eosin (HE)- and immunofluorescent staining (IFS)

Skin necropsies were snap frozen in Tissue-Tek OCT Compound freezing medium (Sakura Finetek USA Inc.) and HE- and IFS performed on ethanol-fixed tissue sections of 6 µm thickness [9], [13]. For detection of MusPV1 L1 protein, sections were stained with a rabbit polyclonal immune serum directed against MusPV1 L1 at a dilution of 1∶4000 and detected with either an Alexa Fluor 488 or an Alexa Fluor 594-conjugated donkey anti-rabbit secondary antibody (both Life Technologies), as indicated. Co-stainings with either directly conjugated Alexa Fluor 488-anti-mouse CD4 or Alexa Fluor 488-anti-mouse CD8a antibodies (both Biolegend; dilution 1∶100) were performed to detect CD4^+^ or CD8^+^ T cells, respectively. To determine localization of MusPV1 L1 in relation to basal keratinocytes, sections were co-stained with a phycoerythrin-conjugated anti-CD49f antibody (integrin alpha 6, BD Biosciences). Nuclei were visualized by mounting sections with ProLong Gold antifade reagent containing 4′,6-diamidino-2-phenylindole (DAPI) (LifeTechnologies). All microscopy analyses were performed on a Zeiss LSM 510 UV system and color levels of images were processed equally in Adobe Photoshop across experiments.

### Preparation of single cell suspensions, blood collection and FACS analyses

Spleens, iliac and inguinal lymph nodes, and tail skin were harvested from CO_2_ euthanized mice. To obtain single cell suspensions, spleens and lymph nodes were enzymatically digested with DNAse I (0.2 mg/ml; Roche) with and without Collagenase A (0.5 mg/ml; Roche), respectively, and processed as described previously [53]. Skin tissues were cut into fine pieces and incubated with collagenase IV (2 mg/ml; Worthington Biochemical Corp.) plus DNAse I (0.1 mg/ml) in RPMI 1640, supplemented with 10% fetal bovine serum and 1% penicilin/streptomycin, for 1 hr at 37°C. All cell suspensions were passed through 70 µM nylon mesh filters (BD Falcon) prior to lysis of erythrocytes with Ammonium-Chloride-Potassium Buffer (ACK; Lonza).

Blood was collected in heparinized tubes and erythrocytes removed by ACK lysis.

After blocking of Fc receptors by incubation with anti-mouse CD16/CD32 antibody (BD Pharmingen), surface-stainings were performed on single cell suspensions from tissues and blood using anti-mouse CD4-PerCP/Cy5.5 (clone RM 4-5; BD Pharmingen) and anti-mouse CD8a-FITC labeled antibodies (clone YTS; Abcam). Cells were fixed with Cytofix/Cytoperm (BD Biosciences) and acquisitions of flow cytometric data performed on a FACSCanto with FACSDiva software (BD Biosciences) [53]. The FlowJo software was used for analyses.

### Statistical analyses

Statistical analyses (Mann Whitney tests) were performed using the GraphPad Prism Software 6.00 for Windows.

## Supporting Information

Figure S1 **(Related to** **Figure 1** **) Cyclosporin A administration promotes strain-dependent MusPV1-induced papilloma formation and lesion maintenance; Lesion size over time after cessation of cyclosporin A.** (A) 129S6/SvEv mice were intermediately susceptible and (B) DBA/2NCr mice resistant to MusPV1-induced papillomatosis while under cyclosporin A treatment. (C) Lesion size over time after cessation of cyclosporin A treatment (corresponding to 4 weeks post-infection) in four representative MusPV1-infected Cr:ORL SENCAR mice. Two representative mice with smaller papillomas of 2 and 4 mm in size after cessation of cyclosporin A treatment (grey and red lines, respectively) and two representative mice with larger papillomas of 9 mm (green and blue lines) are shown.
(PPTX)

Figure S2 **Evaluation of latency 5 months post-infection in MusPV1-infected mouse strains.** Mice (n = 4 per experimental group) previously inoculated with 6×10^10^ MusPV1 virions per animal were subjected to cyclosporin A administration at 4 months post-infection for a period of 4 weeks. After this period (corresponding to 5 months post-infection) mice did not develop visible lesions. Both, MusPV1 E1∧E4 spliced transcripts and the viral genome were undetectable in skin tissues taken from the inoculation sites. Absolute copy numbers of the MusPV1 genome, when detectable, in these samples are shown as numbers above each bar. As controls, skin tissues harvested 4 weeks post-infection from cyclosporin A-treated/MusPV1-infected Cr:ORL SENCAR mice (n = 4) were included in the analysis (mean ± SEM shown).
(PPTX)

Figure S3 **Transient papilloma development after inoculation with 1×10^12^ MusPV1 virions in Cr:ORL SENCAR mice.** (A) Small transient papillomas developed 2–3 weeks after infection with 1×10^12^ MusPV1 in Cr:ORL SENCAR mice. One representative mouse at week 3 post-infection shown. (B) The lesions showed histological features consistent with papillomas. Hematoxylin-eosin stained tissue section (magnification 4×) of a representative mouse. (C) Determination of MusPV1-specific E1∧E4 spliced transcripts relative to beta-actin revealed low, but detectable amounts of E1∧E4 in the papillomas at 3 weeks after infection with 1×10^12^ MusPV1 virions (M), which were absent in mock-infected littermates (0). Data from one representative mouse per group are shown; real time PCR reactions were performed in triplicate (mean ± SEM shown). (D) Immunofluorescent staining of a papilloma taken 3 weeks post-infection revealed punctate, cytoplasmic MusPV1 L1 staining (green, detection with an Alexa Fluor 488-labeled secondary antibody) in the basal and lower spinous layers, and nuclear L1 staining in the upper spinous and granular layers of the epithelium. A phycoerythrin-conjugated anti-CD49f antibody (red) was used for co-staining of basal keratinocytes to faciliate orientation. (E) Skin tissues taken from the tail skin of a mock-infected littermate showed anti-CD49f staining, but lacked MusPV1 L1 staining. (F) The transient papillomas of Cr:ORL SENCAR mice contained infectious MusPV1 virions that were able to induce papilloma formation on the tail of an athymic nude NCr mouse after experimental transmission. (G) C57BL/6 mice did not develop papillomas after inoculation with 1×10^12^ MusPV1 virions (representative mouse at 3 weeks post-infection shown).
(PPTX)

Figure S4 **Monitoring of CD4^+^ and CD8^+^ T cell depletion in Cr:ORL SENCAR mice.** Flow cytometry analyses were performed at indicated time points in the peripheral blood of (A) CD4- and (B) CD8-depleted MusPV1-infected Cr:ORL SENCAR mice and verified the depleted state. (C) Isotype-depleted/MusPV1-infected, (D) non-depleted/MusPV1-infected and (E) mock-infected littermates served as controls.
(PPTX)

Figure S5 **Monitoring of CD4^+^ and CD8^+^ T cell depletion in C57BL/6NCr mice.** At indicated time points during (A) CD3 depletion, (B) single CD4 depletion, (C) single CD8 depletion and (D) combined CD4+8 depletion flow cytometry analyses verified the depleted state in the blood of MusPV1-infected C57BL/6NCr mice. (E) Isotype-depleted/MusPV1-infected, (F) non-depleted/MusPV1-infected and (G) mock-infected littermates served as controls.
(PPTX)

Table S1 **(Transient) papilloma development in immunocompetent Cr:ORL SENCAR mice.** MusPV1 virions were serially diluted (10-fold, ranging from 1×10^8^ to 1×10^12^ MusPV1 virions per inoculation site), and decreasing doses applied to individual immunocompetent Cr:ORL SENCAR mice. After an observation period of 2.5 weeks post-infection mice were evaluated for papilloma formation.
(PPTX)
