# Supplementary figures and images for: Strain-Specific Properties and T Cells Regulate the Susceptibility to Papilloma Induction by Mus musculus Papillomavirus 1
Source: PLoS Pathog. 2014 Aug 14;10(8):e1004314. doi: 10.1371/journal.ppat.1004314 (PMC4133403; doi:10.1371/journal.ppat.1004314)

## Slide 1
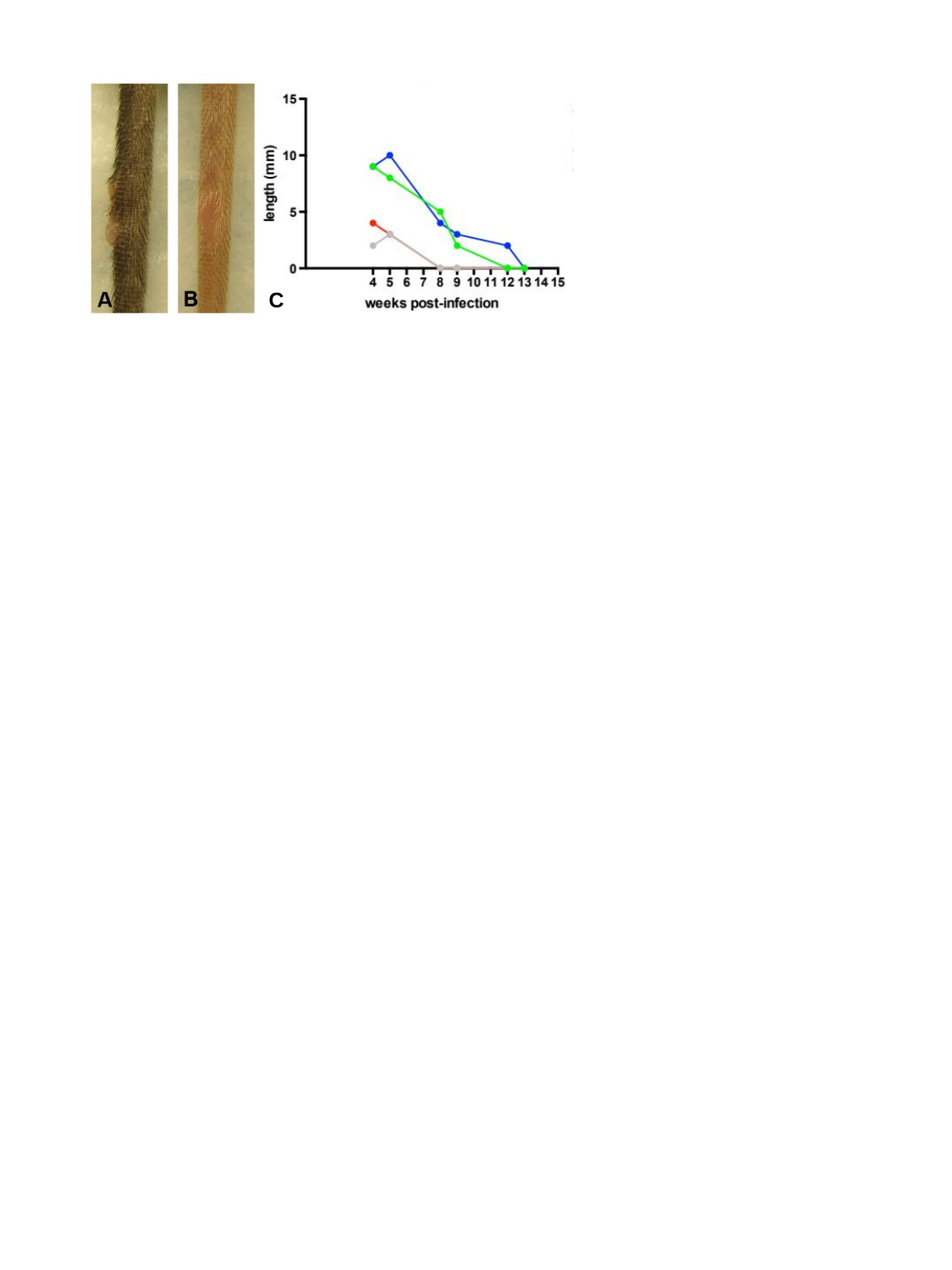

C

Supplement: Figure S1 — (Related to Figure 1 ) Cyclosporin A administration promotes strain-dependent MusPV1-induced papilloma formation and lesion maintenance; Lesion size over time after cessation of cyclosporin A. (A) 129S6/SvEv mice were intermediately susceptible and (B) DBA/2NCr mice resistant to MusPV1-induced papillomatosis while under cyclosporin A treatment. (C) Lesion size over time after cessation of cyclosporin A treatment (corresponding to 4 weeks post-infection) in four representative MusPV1-infected Cr:ORL SENCAR mice. Two representative mice with smaller papillomas of 2 and 4 mm in size after cessation of cyclosporin A treatment (grey and red lines, respectively) and two representative mice with larger papillomas of 9 mm (green and blue lines) are shown. (PPTX) [file ppat.1004314.s001.pptx]

## Slide 1
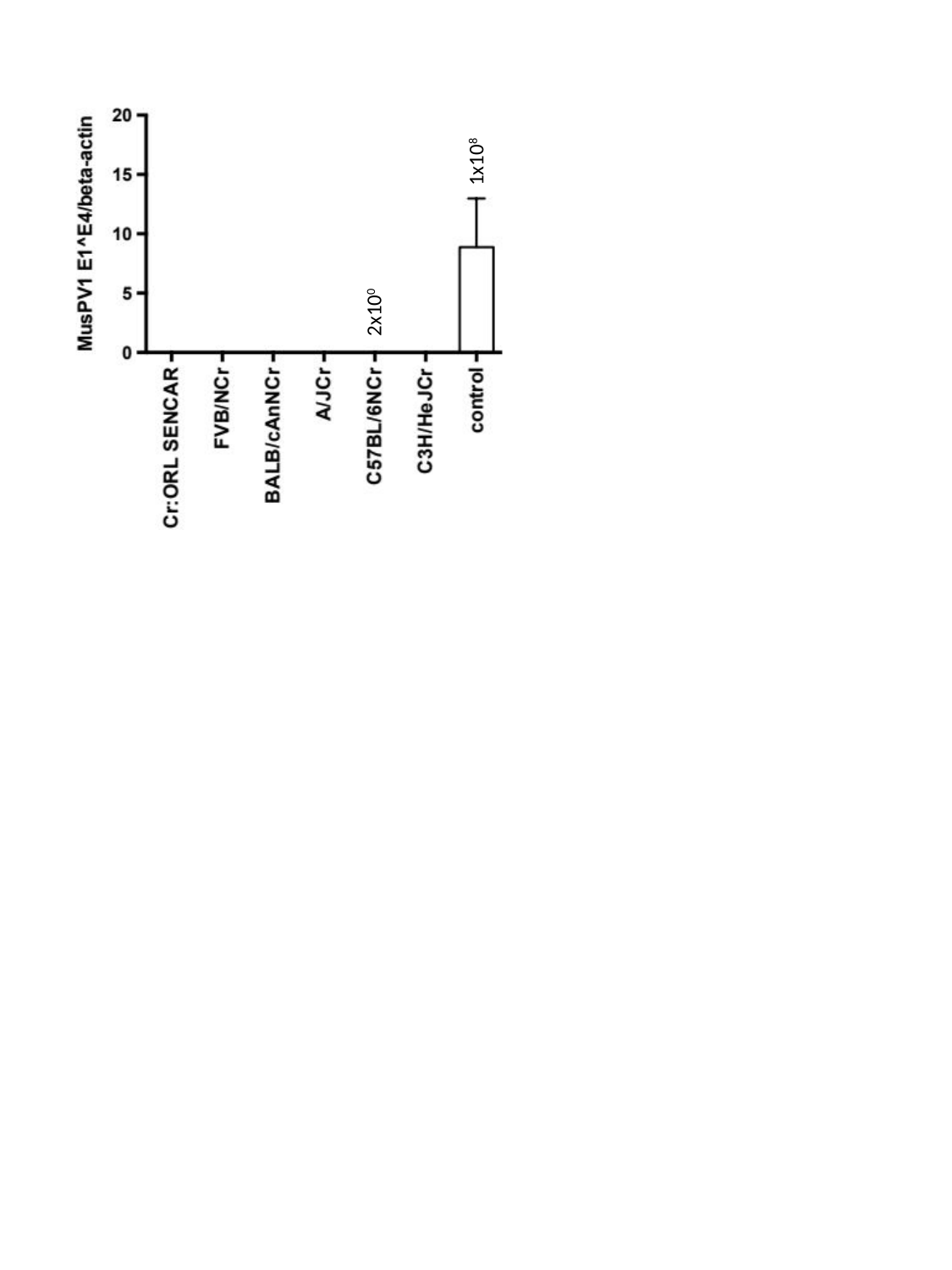

1x108
2x100

Supplement: Figure S2 — Evaluation of latency 5 months post-infection in MusPV1-infected mouse strains. Mice (n = 4 per experimental group) previously inoculated with 6×10^10^ MusPV1 virions per animal were subjected to cyclosporin A administration at 4 months post-infection for a period of 4 weeks. After this period (corresponding to 5 months post-infection) mice did not develop visible lesions. Both, MusPV1 E1∧E4 spliced transcripts and the viral genome were undetectable in skin tissues taken from the inoculation sites. Absolute copy numbers of the MusPV1 genome, when detectable, in these samples are shown as numbers above each bar. As controls, skin tissues harvested 4 weeks post-infection from cyclosporin A-treated/MusPV1-infected Cr:ORL SENCAR mice (n = 4) were included in the analysis (mean ± SEM shown). (PPTX) [file ppat.1004314.s002.pptx]

## Slide 1
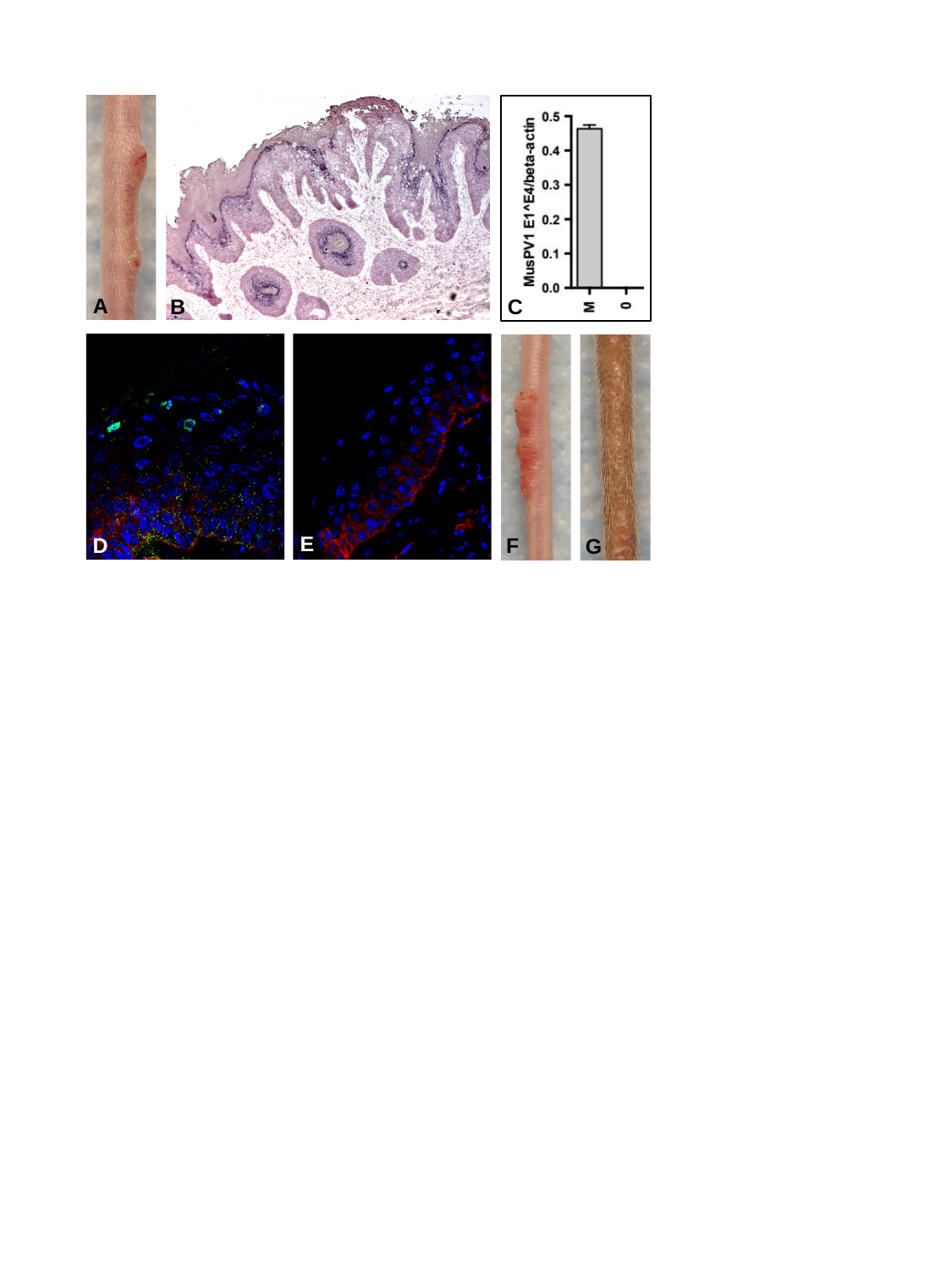

A
C
B
E
D
F
G

Supplement: Figure S3 — Transient papilloma development after inoculation with 1×10^12^ MusPV1 virions in Cr:ORL SENCAR mice. (A) Small transient papillomas developed 2–3 weeks after infection with 1×10^12^ MusPV1 in Cr:ORL SENCAR mice. One representative mouse at week 3 post-infection shown. (B) The lesions showed histological features consistent with papillomas. Hematoxylin-eosin stained tissue section (magnification 4×) of a representative mouse. (C) Determination of MusPV1-specific E1∧E4 spliced transcripts relative to beta-actin revealed low, but detectable amounts of E1∧E4 in the papillomas at 3 weeks after infection with 1×10^12^ MusPV1 virions (M), which were absent in mock-infected littermates (0). Data from one representative mouse per group are shown; real time PCR reactions were performed in triplicate (mean ± SEM shown). (D) Immunofluorescent staining of a papilloma taken 3 weeks post-infection revealed punctate, cytoplasmic MusPV1 L1 staining (green, detection with an Alexa Fluor 488-labeled secondary antibody) in the basal and lower spinous layers, and nuclear L1 staining in the upper spinous and granular layers of the epithelium. A phycoerythrin-conjugated anti-CD49f antibody (red) was used for co-staining of basal keratinocytes to faciliate orientation. (E) Skin tissues taken from the tail skin of a mock-infected littermate showed anti-CD49f staining, but lacked MusPV1 L1 staining. (F) The transient papillomas of Cr:ORL SENCAR mice contained infectious MusPV1 virions that were able to induce papilloma formation on the tail of an athymic nude NCr mouse after experimental transmission. (G) C57BL/6 mice did not develop papillomas after inoculation with 1×10^12^ MusPV1 virions (representative mouse at 3 weeks post-infection shown). (PPTX) [file ppat.1004314.s003.pptx]

## Slide 1
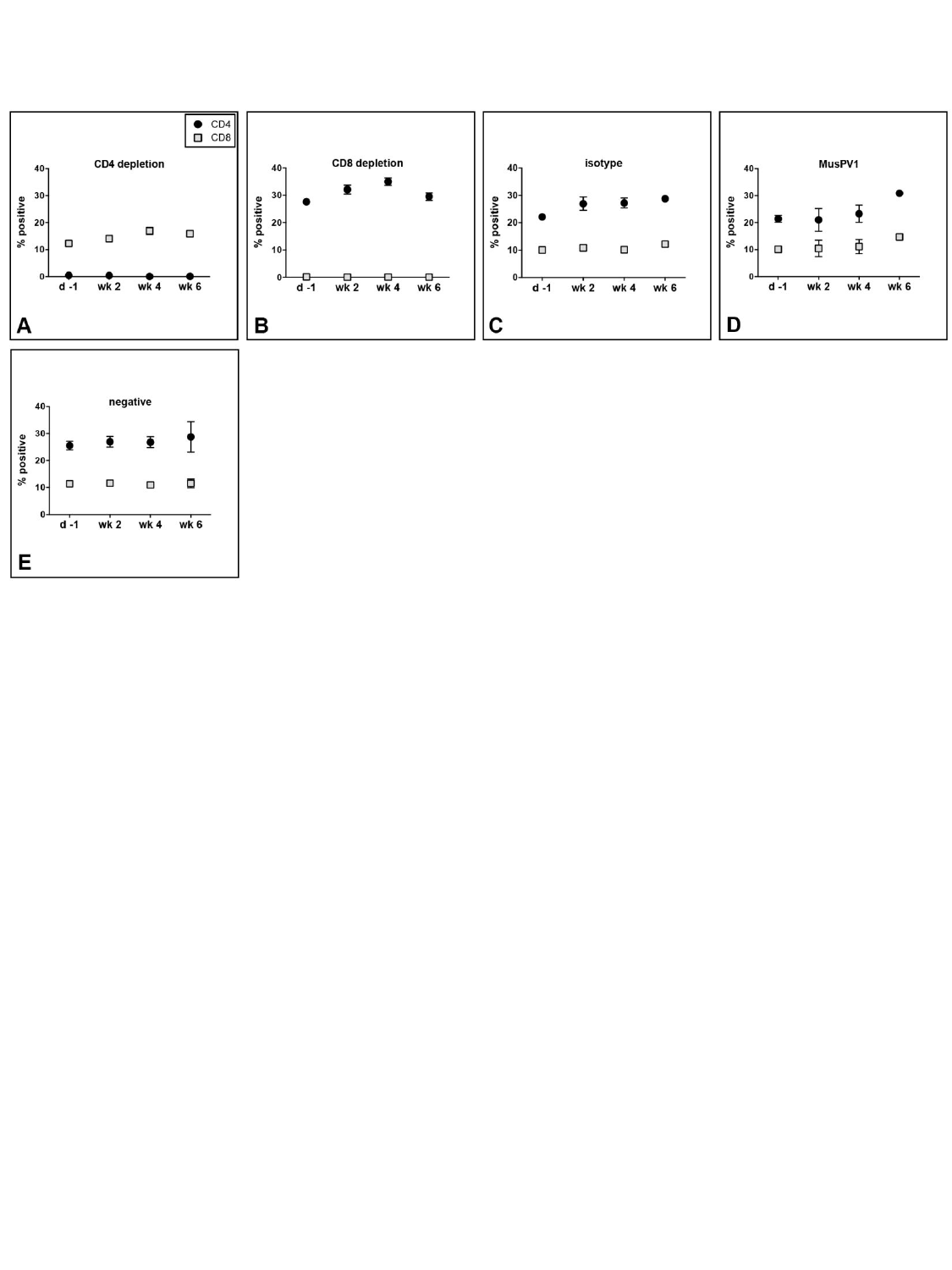

Supplement: Figure S4 — Monitoring of CD4+ and CD8+ T cell depletion in Cr:ORL SENCAR mice. Flow cytometry analyses were performed at indicated time points in the peripheral blood of (A) CD4- and (B) CD8-depleted MusPV1-infected Cr:ORL SENCAR mice and verified the depleted state. (C) Isotype-depleted/MusPV1-infected, (D) non-depleted/MusPV1-infected and (E) mock-infected littermates served as controls. (PPTX) [file ppat.1004314.s004.pptx]

## Slide 1
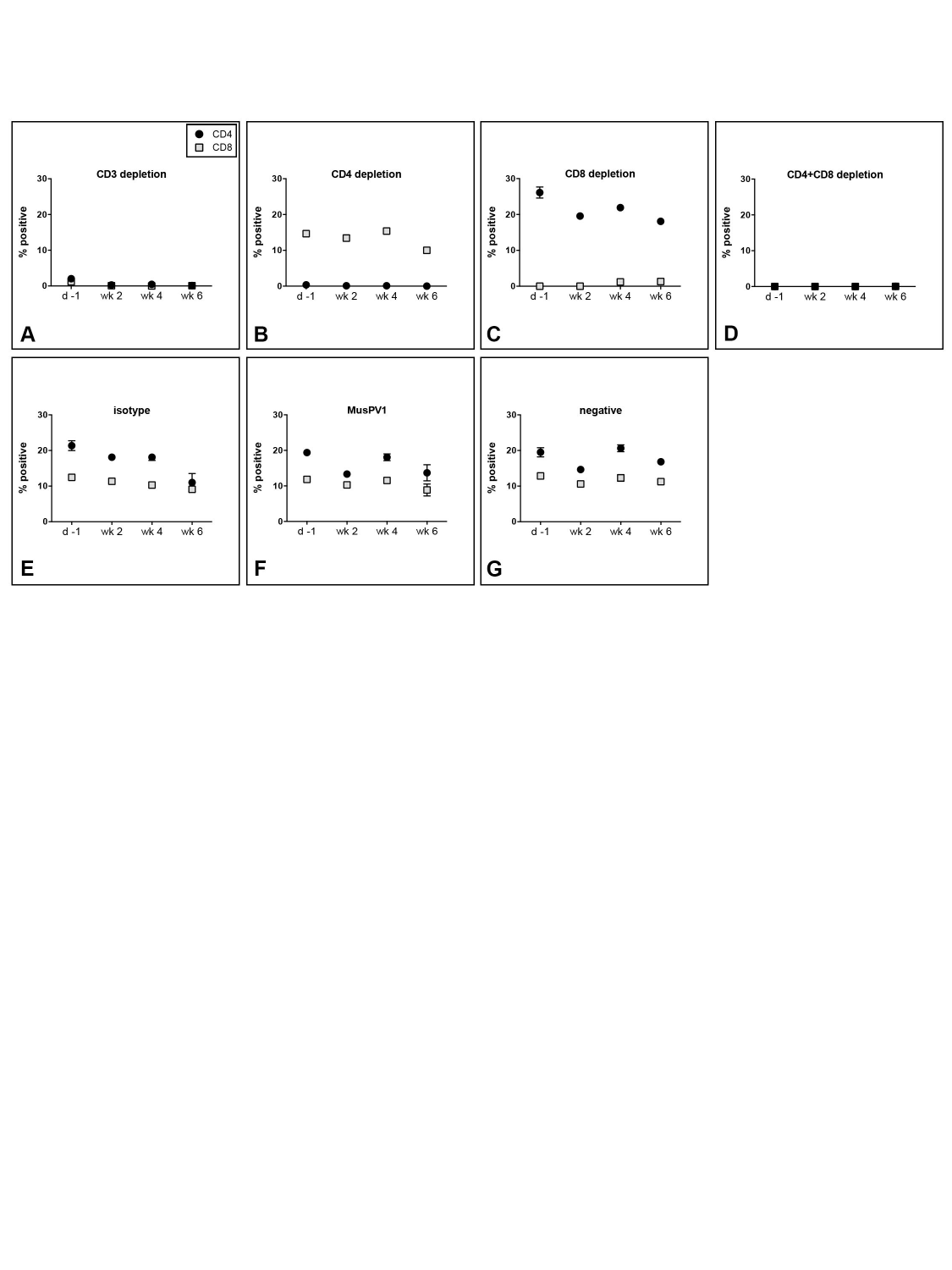

Supplement: Figure S5 — Monitoring of CD4+ and CD8+ T cell depletion in C57BL/6NCr mice. At indicated time points during (A) CD3 depletion, (B) single CD4 depletion, (C) single CD8 depletion and (D) combined CD4+8 depletion flow cytometry analyses verified the depleted state in the blood of MusPV1-infected C57BL/6NCr mice. (E) Isotype-depleted/MusPV1-infected, (F) non-depleted/MusPV1-infected and (G) mock-infected littermates served as controls. (PPTX) [file ppat.1004314.s005.pptx]
