# Supplementary material for: Strain-Specific Properties and T Cells Regulate the Susceptibility to Papilloma Induction by Mus musculus Papillomavirus 1
Source: PLoS Pathog. 2014 Aug 14;10(8):e1004314. doi: 10.1371/journal.ppat.1004314 (PMC4133403; doi:10.1371/journal.ppat.1004314)
Supplement: Table S1 — (Transient) papilloma development in immunocompetent Cr:ORL SENCAR mice. MusPV1 virions were serially diluted (10-fold, ranging from 1×10^8^ to 1×10^12^ MusPV1 virions per inoculation site), and decreasing doses applied to individual immunocompetent Cr:ORL SENCAR mice. After an observation period of 2.5 weeks post-infection mice were evaluated for papilloma formation. (PPTX) [file ppat.1004314.s006.pptx]

## Slide 1
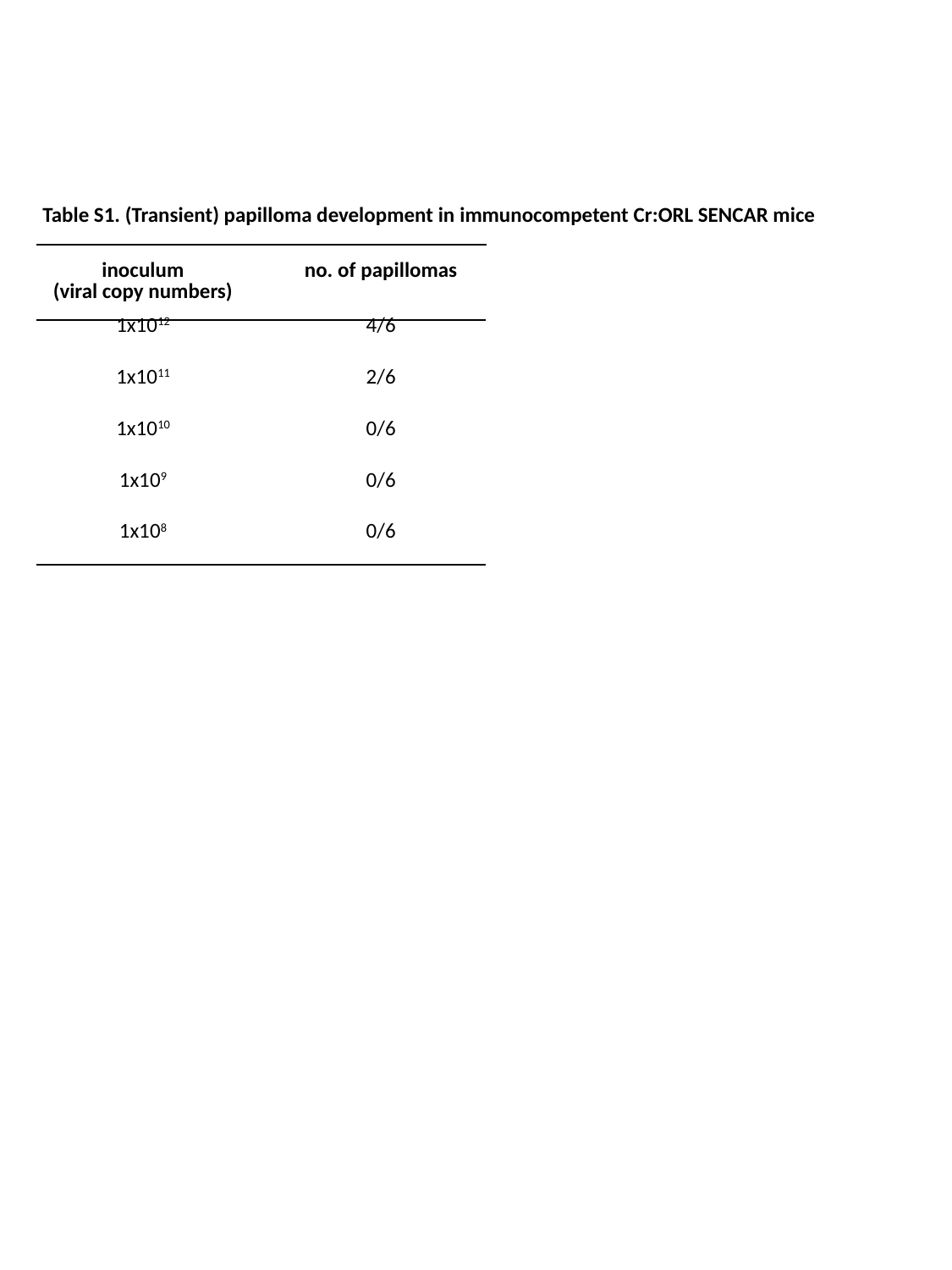

Table S1. (Transient) papilloma development in immunocompetent Cr:ORL SENCAR mice
| inoculum (viral copy numbers) | no. of papillomas |
| --- | --- |
| 1x10^12^ | 4/6 |
| 1x10^11^ | 2/6 |
| 1x10^10^ | 0/6 |
| 1x10^9^ | 0/6 |
| 1x10^8^ | 0/6 |
